# The vestibular outcomes in non-blast related traumatic brain injury and the role of severity, aetiology and gender: a scoping review

**DOI:** 10.3389/fneur.2025.1654850

**Published:** 2026-01-20

**Authors:** Kübra Bölükbaş, Laura Edwards, Olivia R. Phillips, Veronica Kennedy, Kathryn Fackrell

**Affiliations:** 1 Hearing Sciences, Division of Mental Health and Clinical Neuroscience, School of Medicine, University of Nottingham, Nottingham, United Kingdom; 2 National Institute of Health and Social Research (NIHR) Nottingham Biomedical Research Centre, Nottingham, United Kingdom; 3 Division of Rehabilitation Medicine, University Hospitals of Derby and Burton NHS Foundation Trust, Derby, United Kingdom; 4 Injury, Inflammation and Recovery Sciences, School of Medicine, University of Nottingham, Nottingham, United Kingdom; 5 Lifespan and Population Health, School of Medicine, University of Nottingham, Nottingham, United Kingdom; 6 Department of Paediatric Audiology, Bolton NHS Foundation Trust, Bolton, United Kingdom

**Keywords:** aetiology, BPPV, dizziness, gender, TBI severity, traumatic brain injury, vertigo, vestibular

## Abstract

**Introduction:**

Traumatic brain injury (TBI) can lead to various vestibular impairments. This review explored common vestibular outcomes associated with non-blast related TBI and examined possible differences in vestibular outcomes based on TBI severity, aetiology, and gender.

**Methods:**

A scoping review was conducted using an established methodological framework, which involved electronic and manual searches of databases and journals. Records published in English were included which focused on vestibular outcomes and assessments associated with non-blast related TBI in individuals 18 years and older. Out of a total of 19.200 records, 50 met the inclusion criteria. Data were collated and categorised based on the objectives of the research.

**Results:**

Benign paroxysmal positional vertigo (BPPV) was found in 38% of 50 studies. Furthermore, despite normal peripheral vestibular function, central processing disorders such as impaired self-motion perception and sensory integration dysfunction were also observed in TBI patients. TBI severity did not have a consistent effect on vestibular outcomes, while in terms of aetiology BPPV was observed to be more common in falls related TBI. Gender differences in vestibular findings were limited and varied across studies.

**Conclusion:**

The complex nature of TBI, combined with the intricate structure of the vestibular system, makes it difficult to establish a clear framework on the vestibular outcomes following TBI. Additionally, the use of different vestibular assessment methods across studies and the inconsistent reporting of outcomes complicates the holistic analysis of the data. Therefore, in order to better understand and manage the effects of TBI on the vestibular system, it is crucial to develop standardised clinical practices and assessment guidelines.

## 1 Introduction

Traumatic Brain Injury (TBI) is a widespread cause of death and disability worldwide (1, 2). TBI, with its associated physical, behavioural, cognitive and emotional deterioration (3–5), not only adversely impacts the quality-of-life of individuals but also causes a global burden on states due to the costs it incurs (6). It is estimated that the annual cost of TBI in the United Kingdom (UK) alone is 15 billion pounds (7).

Different methods are used for classifying the severity of TBI. Commonly, the Glasgow Coma Scale (GCS) at the time of injury and the duration of post-traumatic amnesia are employed for classification of TBI as mild (GCS: 13–15; post-traumatic amnesia: <24 h), moderate (9–12; >24 h), or severe (3–8; >7 days) (8). For TBI cases presenting to the hospital, more than 90% are classified as mild TBI (9). The common causes (aetiologies) known to lead to TBI include motor vehicle accidents (MVA), falls, sports-related injuries, assaults (all of these also known as non-blast related) and explosions (blast-related). The mechanisms of brain injury can vary across different aetiologies, particularly between blast-related and non-blast related TBI. In blast-related TBI, damage occurs due to the high-pressure waves generated during the explosion, whereas in non-blast related TBI, damage is typically observed as a result of a blunt force trauma to the head, concussion, or penetration (10). Due to this fundamental distinction and the different damage that would be related to distinct vestibular issues, the focus of this review will be on non-blast related TBI.

Dizziness, vertigo and imbalance stand out as some of the most commonly reported issues associated with TBI. The prevalence of dizziness and/or vertigo post-TBI was found to be between 23.8 and 81% (11, 12). The unpredictable damage caused by TBI to the brain can complicate the detection of the exact source of resulting vertigo and/or dizziness complaints. However, anatomically, TBI can lead to these symptoms by causing damage to the peripheral and central vestibular systems, brainstem pathways, as well as visual, motor, and oculomotor pathways (13). For example, currently, the most commonly reported peripheral vestibular disorder post-TBI is Benign Paroxysmal Positional Vertigo (BPPV) (14–16).

Despite the availability of many studies related to vestibular outcomes associated with TBI, there is a need for a comprehensive review synthesising common vestibular findings related to non-blast related TBI, including the relationship of TBI aetiology, severity and gender with vestibular conditions. A review conducted for this purpose can contribute to the development of diagnostic and treatment methods in this patient group, helping to identify appropriate and effective strategies for addressing vestibular complaints. Thus, a wide range of benefits can be achieved in the long term, from improving individual quality-of-life to alleviating the economic burden on states.

The aim of this study is to map and synthesise the literature on vestibular impairments associated with non-blast related TBI, with specific consideration of the potential influence of injury severity, aetiology, and gender.

In particular, the research questions are:

i) What are the common vestibular assessments and impairments of TBI,
ii) Whether vestibular outcomes vary according to severity of TBI,
iii) Whether vestibular outcomes vary according to aetiology of TBI,
iv) Whether vestibular outcomes vary by gender following TBI.

To address these broad and diverse research questions, map the literature, summarise the findings, and synthesise evidence from more than one study design, a scoping review was selected as the most appropriate approach (17, 18).

## 2 Materials and methods

This scoping review followed the 6-stages framework developed by Arksey and O’Malley (18). These stages were conducted in the following order: (1) identifying the research question(s), (2) identifying the relevant studies using appropriate keywords, (3) selecting relevant studies through an iterative scanned title, abstract, and full text, (4) extraction and charting the data, (5) collating, summarising and reporting of the results, (6) clinician review. The review was conducted in accordance with the PRISMA-S guidelines (19) (see Supplementary Appendix Table 1 for PRISMA-ScR Checklist).

### 2.1 Identifying the research question(s)

The research questions (listed above) were developed collaboratively with team members based on existing knowledge and literature in the field.

### 2.2 Identifying relevant studies

#### 2.2.1 Eligibility criteria

To be included, records had to report studies involving adults (≥18 years old) with non-blast related TBI, focusing on vestibular outcomes and assessments. This included self-reported vestibular outcomes if vestibular impairment was clinically confirmed. If articles reported both auditory and vestibular outcomes, only the vestibular components were included. Records were eligible if they reported symptoms or assessment pre-treatment and were sourced from cohort studies, randomised control trials, case series, and case studies, as well as from grey literature such as dissertations and theses. In studies where treatments (e.g., Epley or Semont manoeuvre) were applied, the follow-up assessments were not reported in this review. Only initial (pre-treatment) assessments, including any follow-up conducted before treatment were reported. All records included in the study were published in English language and were available in full text. Cases that did not meet our inclusion criteria were removed from the case series studies.

Records were excluded if the studies were reporting adults who may have experienced blast-related TBI, whiplash injuries or non-TBI conditions (e.g., strokes, acoustic neuroma), TBI in childhood or with pre-existing audio-vestibular disorders prior to TBI, or where the aetiology of TBI was unspecified. Records were also excluded if they did not clearly define TBI or did not report structural injury or functional impairment resulting from TBI or only reported auditory condition without any assessment of vestibular outcomes or broadly focused on balance assessments rather than vestibular-specific tests. Records reporting the reliability and validity of tests, animal studies, review articles, including systematic reviews, book chapters, qualitative research studies and any sources presenting protocols, personal/expert opinions or tutorials were excluded.

#### 2.2.2 Search strategy

The research strategy was developed by the research team and was supported by a medical information specialist (Dr Farhad Shokraneh). The search was conducted following Cochrane Handbook (20) and Cochrane’s MECIR (21) and PRESS guideline for peer-reviewing the search strategies (22). Electronic databases were searched, including Embase, MEDLINE, ProQuest Dissertations & Theses A&I, PsycINFO, Science Citation Index Expanded and SPORTDiscus. Initial searches were conducted in May 2022. An additional database search of Cochrane Central Register of Controlled Trials (CENTRAL) was conducted in November 2025. The search strategy included keywords on TBI, auditory and vestibular conditions (a separate review was conducted for auditory outcomes). These were reviewed and revised following a primary search (see Supplementary Appendix Table 2 for search strategy). Specific search term strategies were employed across each search engine, covering article topics, titles, abstracts, and keywords. Filters were implemented to select articles written in English language and involving human participants only, when feasible. No limitations were imposed on the search timeframe. Additionally, manual searches of reference lists and prominent journals, identified using the interquartile rule for outliers, were conducted to identify additional eligible documents. The final database and manual searches were conducted in November 2025.

### 2.3 Study selection

Records retrieved from electronic searches were transferred to EndNote (version X9), containing citation, title, and abstract, where duplicates were eradicated. Four researchers (KB, KF, LE, OP) independently screened the records via Rayyan (23), initially scrutinizing the title and abstract, followed by a review of the full text. Lead researcher (KB) was responsible for screening all records. The records obtained as a result of the manual search were subjected to full-text screening. In instances of discordance regarding the eligibility of any record, reviewers deliberated on their reasons until an agreement was reached, or a third reviewer (VK) was consulted to achieve a majority decision.

### 2.4 Extraction and charting of the data

A data extraction form was created and developed in Microsoft Excel and piloted on five included records and was subsequently modified following team discussions. Data from each record was extracted by the lead researcher (KB) and checked by KF. Data were extracted on study characteristics, study population, TBI characteristics, vestibular complaints and assessments/outcomes, and limitations (Box 1).

**BOX 1 Data extraction fields**

**Table:** 

Authors
Year of publication
Country where study was conducted
Study title
Aim of study
Study design
Study population
Sample size
Age
Gender
Classification method for TBI
Severity of TBI
Causes/aetiology of TBI
Status pre/post-TBI
Presence of coma
Radiological results
List of vestibular complaints
List of vestibular assessment tools
Vestibular outcomes
Assessment time since injury
Single or repeated assessments
Study limitations

### 2.5 Collating, summarising and reporting results

Extracted data were collated and categorised depending on the objectives of our research. Each relevant study was grouped according to categories such as vestibular outcomes, severity of TBI, aetiology, and gender effects. Data were then summarised to highlight common patterns and significant variations in vestibular outcomes.

### 2.6 Clinician review

Following the identification of categories, categorised findings were reviewed by clinicians (LE & VK).

## 3 Results

**Figure 1:**
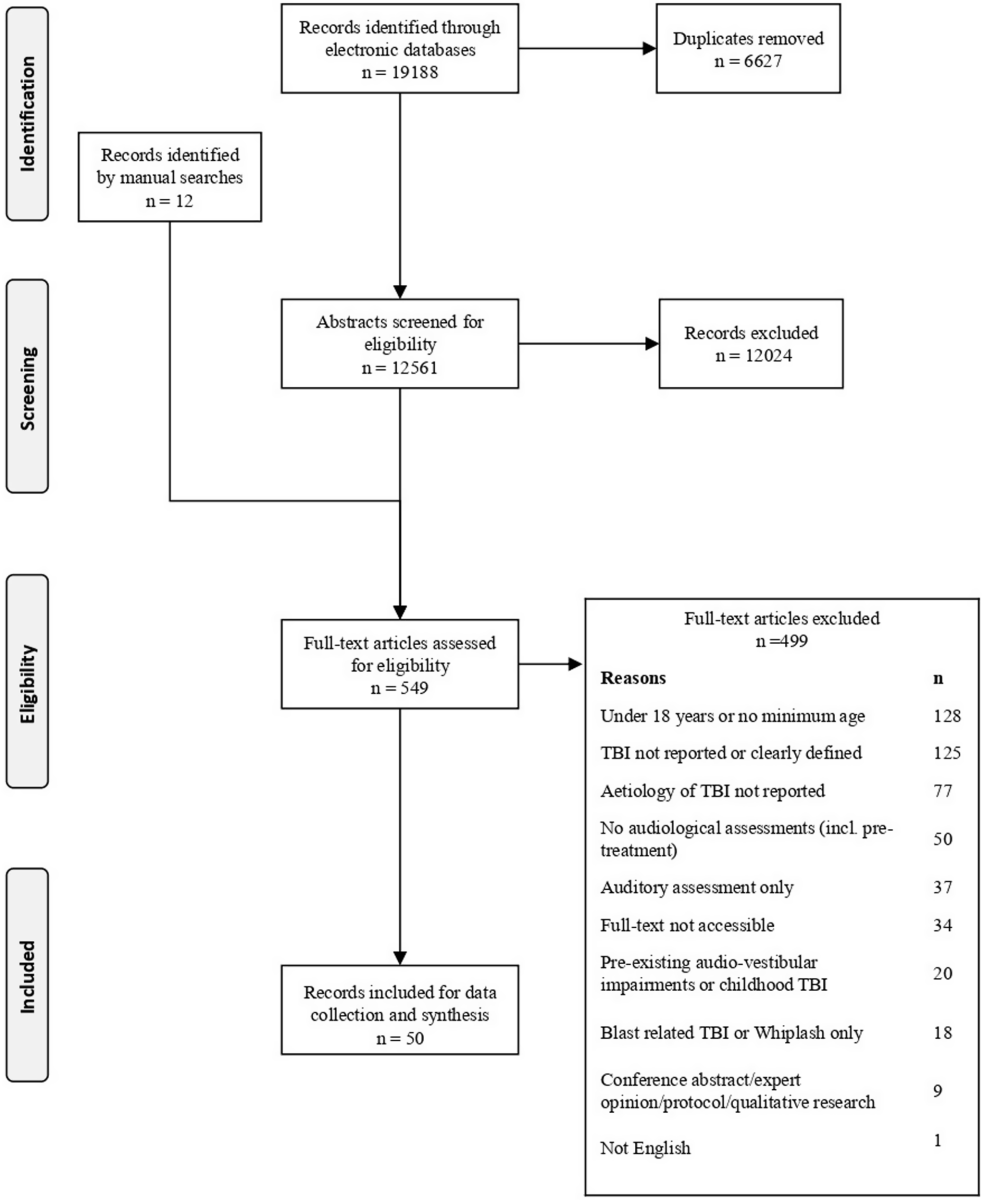
The PRISMA flow diagram for the study selection process.

The process of record identification and selection is in the PRISMA flow diagram (Figure 1). Electronic searches were produced in an initial set of 19,188 records. After removing duplicates, 12,561 records remained and of these, 12,024 were excluded during the title and abstract screening due to not meeting the eligibility criteria. Manual searches identified a further 12 potential articles which were subjected to full-text screening. Of the remaining 549 records, a further 499 records were excluded at the full-text screening. Most commonly the studies excluded did not report TBI or clearly define TBI, included participants under 18 years old and did not report TBI aetiology. Full-text records could not be located for 31 records. None of these records could be traced, regardless of support from the University of Nottingham librarian. The electronic and manual searches resulted in a final list of 50 eligible full-text records for data collection.

### 3.1 Study characteristics

Table 1 summarises the characteristics of the study and participants. Among the 50 included articles, the most commonly reported study design was case report(s)/case series (27/50, 54%) (24–50). The other study designs are shown in Table 1. Articles were published from 1956 to 2024. Studies were mainly conducted in the United States (n = 19), followed by the UK (*n* = 4) and Australia (*n* = 4) (Table 1).

**Table 1:** Characteristics of included studies.

Ref	Country	Study design	Research aim	Sample size and age range (years)	Gender	Severity of TBI	Criteria of severity	Fall	MVA	Assault/direct impact	Sports injury	Other	Time of audiological assessment	Pre-TBI status
Feneley and Murthy (1994) (24)	UK	Case report	To describe the case who presented with acute bilateral deafness and vestibular dysfunction following occipital bone fracture	1 (57 yrs)	M	NR	NR	✓					3 dys (f/u: 3 wks)	Excellent health, with no meds or history of excessive alcohol consumption
Bertholon et al. (2005) (25)	France	Case report	To report cases who complained of positional vertigo shortly after head trauma	1: Case 1 (19 yrs)*	M	NR	NR	✓					1 mth	C1: No significant medical history
Kagoya et al. (2010) (26)	Japan	Case report	To present a very rare case of stapedial footplate fracture in which the superstructure with part of the footplate was dislocated and adhered to the tympanic membrane	1 (25 yrs)	F	NR	NR		✓				11 mths	Unremarkable medical history
Ylikoski et al. (1982) (27)	USA	Case report	To search for pathologic changes indicating nerve injury by examining the operative specimens of the eighth nerve from patients with post-traumatic dizz. and combining these findings with the clinical, otologic and surgical features of each case, to determine the site of primary lesion	2: Cases 8, 9 (55, 53 yrs)*	M	C8: NR C9: NR	NR		✓ (2)				NR	NR
Roup et al. (2020) (28)	USA	Case report	To present a case report of a patient with a history of TBI, including self-perceived hearing difficulties and poorer-than-normal auditory processing performance	1 (58 yrs)	F	Mild	NR		✓				12 mths	No hearing or listening problems
Jacobs et al. (1979) (47)	USA	Case report	To present results of surgical repair in three patients with fistulas	1: Case 1 (59 yrs)*	F	C1: NR	NR					✓	Subsequent mths	NR
Herdman (1990) (49)	USA	Case studies	Some common vestibular deficits that occur with a head injury will be illustrated, and the test results, exercise treatment, and course of recovery in those patients will be described	3: Cases 1–3 (53, 39, 40 yrs)	M = 2 F = 1	NR	NR	✓ (2)	✓				C1: 5 dys (f/u: 2 yrs) C2: 10 wks C3: 12 dys f/u: 4 mths)	NR
Jani et al. (1991) (35)	USA	Case report	To report the usefulness of magnetic resonance imaging and auditory brainstem evoked responses in diagnosis	1 (46 yrs)	F	Mod. or Severe	NR			✓			13th dy	History of major mood disorder
Fitzgerald (1995) (29)	USA	Case report	To discuss the typical history and diagnostic tests for patients with perilymphatic fistula	1: Case 1 (28 yrs)*	F	NR	NR		✓				6 dys (f/u: 10 wks)	NR
Lyos et al. (1995) (42)	USA	Case report	To describe three patients with transverse temporal bone fracture who presented with residual auditory function only to develop profound SNHL	3: Cases 1–3 (20, 20, 26 yrs)	M	NR	NR	✓		✓		✓	C1: 1 wk. C2: 3 mths C3: 5 dys	NR
Johnson (2009) (50)	USA	Case report	NR	1 (47 yrs)	F	Mild	NR	✓					6 mths	NR
Waninger et al. (2014) (37)	USA	Case report	To describe a unique mechanism of ear barotrauma (intratympanic haemorrhage) and concussion caused by helmet-to-helmet contact in American football	1 (26 yrs)	M	Concuss^	NR				✓		36 h	No history of prev. Concuss^ or head/ear injuries
Blackard et al. (2020) (34)	USA	Case report	NR	1 (22 yrs)	F	Concuss^	NR					✓	5 dys (f/u: 1 mth)	No prior history of concuss^
Schuknecht and Davison (1956) (36)	Canada	Case report	NR	1: Case 3 (29 yrs)*	M	NR	NR		✓				2 yrs	NR
Paxman et al. (2018) (43)	Canada	Case report	This case highlights the use of repetitive transcranial magnetic stimulation (rTMS) as a novel treatment option for patients who suffer from post-concussive symptoms and chronic dizz. Secondary to mTBI	1 (61 yrs)	M	Mild	GCS: 15		✓				NR	NR
Ottaviano et al. (2009) (32)	Italy	Case report	To report two cases of SNHL with BPPV and anosmia following traumatic head injury	1: Case 2 (57 yrs)*	F	NR	NR		✓				7 mths	NR
Ralli et al. (2010) (30)	Italy	Case report	The cases of suffering from vertigo after that fell from a camel during a visit to the middle east are described	1: Case 1 (60 yrs)*	M	NR	NR	✓					10 dys	NR
Kanavati et al. (2016) (33)	UK	Case report	NR	1 (24 yrs)	M	NR (GCS: 12)	NR			✓			NR	NR
Preber and Silversklöld (1957) (38)	Sweden	Case report	NR	4: Cases 1–3, 5 (36, 48, 57, 53 yrs)*	M = 2 F = 2	NR	NR	✓	✓ (3)				C1: 1 mth (f/u: 1/2/4 mths) C2: 3 mths (f/u: 3 yrs) C3: 3 mths C5: 2 mths (f/u: 1 yr./ 1.2 yrs)	NR
Tonkin and Fagan (1975) (44)	Australia	Case report	The case histories of thirteen patients with such a fistula are described	3: Cases 7, 9, 10 (20, 55, 26 yrs)*	M	NR	NR	✓ (3)					C7: several wks C9: 5 mths C10: NR	C9: Diabetic underwent a right below the-knee amputation
Lerut et al. (2007) (40)	Belgium	Case report	To discuss the case and the final diagnosis of carotico-cavernous fistula	1 (68 yrs)	F	NR	NR	✓					5 dys (f/u: 2 mths)	NR
Fujimoto et al. (2007) (31)	Japan	Case report	To report a rare and informative case of bilateral progressive SNHL after traumatic subarachnoid haemorrhage and brain contusion, in which cochlear implantation was very successful.	1 (55 yrs)	M	NR	NR	✓					1 mth (f/u: 23 mths)	No history of administration of ototoxic agents, including aminoglycosides
Mohd Khairi et al. (2009) (45)	Malaysia	Case report	To illustrate patients who sustained extradural haemorrhage following a motor vehicle accident with profound SNHL on the opposite ear	1: Case 1 (31 yrs)*	M	NR	NR					✓	NR	NR
Chung et al. (2011) (48)	Korea	Case report	To present the case with bilateral otic capsule violating temporal bone fractures due to head trauma	1 (44 yrs)	M	NR	NR	✓					6 wks	NR
Durbec et al. (2012) (41)	France	Case report	NR	1 (22 yrs)	M	NR	NR			✓			8 dys	NR
Sousa Menezes et al. (2019) (39)	Portugal	Case report	To report the case of a patient with pneumolabyrinth, involving both the vestibule and the cochlea with intense vestibular symptoms, in whom the anatomic defect was evident on surgical exploration and successfully managed surgically	1 (52 yrs)	M	NR	NR	✓					3 dys	No relevant personal history
Kleffelgaard et al. (2016) (46)	Norway	Case series	(i) To describe a grp-based Vestibular Rehabilitation intervention for patients with TBI. (ii) To examine how the intervention may assist in addressing the targeted problems of dizz. and balance problems, by describing changes in self-perceived dizz., balance, and health-related quality-of-life (HRQL)	4: Cases 1–4 (34, 25, 40, 45 yrs)	M = 2 F = 2	Mild	C1: GCS: 15 C2: GCS: 14 C3: GCS 15 C4: GCS 15	✓ (2)	✓	✓			C1: 18 mths C2: 30 mths C3: 9 mths C4: 10 mths	No severe psychological disease, cognitive dysfunction, other comorbidities affecting mobility and independent gain
Taylor et al. (2022) (68)	New Zealand	Retrospective clinical case series	(i) determine how often, and which components of the peripheral vestibular system are affected. (ii) identify characteristics of the injury or clinical features that are associated with peripheral vestibular loss. (iii) explore the relationship between vestibular and oculomotor function and postural stability	99 (18–80 yrs)	M = 40 F = 59	Mild (n95) Mod (n4)	GCS and post-traumatic amnesia	✓ (36)	✓ (17)	✓ (5)	✓ (19)	✓ (22)	Mdn 12 mths	No pre-existing vestibular or neurological diagnoses, and severe visual or musculoskeletal impairment
Ouchterlony et al. (2016) (61)	Canada	Case comparison interventional study	To determine the effectiveness of the canalith repositioning procedure in the treatment of BPPV among patients after mild-to-moderate traumatic brain injury	21 BPPV^1^ (Mdn: 32 yrs) 23 NSD^2^ (Mdn: 36 yrs) 12 No dizz.^3^ (Mdn: 43 yrs)	M = 34 F = 22	Mild (n51) Mod (n5)	GCS Mild: 13–15 Mod: 9–12	✓ (23)	✓(23)	✓ (3)	✓ (5)	✓ (2)	BPPV grp: mdn 50 ± 72.5 dys NSD grp: 65 ± 151 dys No dizz. Grp: 61 ± 50 dys	No significant audio-vestibular signs and cerebrovascular disease
Teramoto et al. (2022) (53)	USA	Retrospective study	To advance the science surrounding female head injury and investigate sex-based differences in concussion assessments among male and female varsity college athletes, strengthened by comprehensive longitudinal assessments following acute injury with baseline comparators	111^a^ (18–24 yrs)	M = 59 F = 52	Concuss^	NR				✓		Pre-session baseline 3 dys	NR
Ahn et al. (2011) (59)	South Korea	Retrospective study	To identify the clinical characteristics of BPPV after TBI and to determine whether clinical differences exist between BPPV after TBI and idiopathic BPPV.	32 (30–74 yrs)	M = 18 F = 14	NR	NR	✓ (8)	✓ (20)	✓ (4)			NR	No history of BPPV, migraine, brain tumour, or cerebrovascular, history of ear disease
Dlugaiczyk et al. (2011) (60)	Germany	Retrospective study	To study the involvement of the different SSCs in post-traumatic BPPV with special reference to anterior canal	1: Case 2 (57 yrs)*	M	NR	NR	✓					3 wks	No serious illness, particularly no vertigo or any kind of inner ear disease
Uyeno et al. (2024) (55)	USA	Retrospective cohort study	To quantify norms and changes in eye-tracking proficiency, and determine vestibular symptom correlations in varsity college athletes following acute mTBI	119 (18–24 yrs)	M = 63 F = 56	Mild	NR				✓		Pre-session baseline, 72 h (f/u: 2^nd^, 3^rd^,4^th^ injury)	66% sustained only 1 injury
Hides et al. (2017) (52)	Australia	Prospective cohort study	To explore changes in sensorimotor function in the acute phase following sports concussion	54 w/ Concuss^ (18–33 yrs)	NR	Concuss^	NR				✓		Pre-session baseline: 3–5 dys	NR
Joseph et al. (2021) (51)	USA	Prospective cohort study	To compare performance on the SOT vestibular score versus the Dual-Task test in individuals with and without subjective balance problems at least 1 yr. after a TBI	26 Symptomatic TBI^4^ 24 Asymptomatic TBI^5^ (21–71 yrs)	M = 31 F = 19	Mild (n24) Mod (n21) Severe (n5)	Department of defence TBI rating scale (110)	✓ (18)		✓ (16)		✓ (16)	Symptomatic TBI: Avg. 584.5 dys Asymptomatic TBI: Avg. 725.4 dys	No major neurological, visual, or autonomic disorders
Motin et al. (2005) (58)	Israel	Prospective study	To identify patients with BPPV among patients with severe TBI and to evaluate the effectiveness of the Particle Repositioning Manoeuvre	20 (19–61 yrs)	M = 18 F = 2	Severe (n20)	NR	✓ (6)	✓ (4)				Mean 67 ± 14 dys	No vertigo any history of inner ear disease
Glendon et al. (2021) (54)	UK	Prospective cohort study	To explore if Vestibular-ocular-motor impairment corelates with longer Return to Play, symptom burden, neurocognitive performance and academic capability	42 (18.2–25.2 yrs)	M = 25 F = 17	Concuss^	NR				✓		Pre-session baseline: 2 dys	NR
Jafarzadeh et al. (2022) (65)	Iran	Prospective cross-sectional study	The vestibular assessment of patients with persistent symptoms of mTBI by different vestibular tests	21 (16–60 yrs)	M = 20 F = 1	Mild	Loss of consciousness <30 min., GCS: 13–15	✓					118.2 ± 52.5 dys	No history of hearing loss, vertigo, imbalance or gait abnormality
McCormick et al. (2023) (64)	USA	Prospective cohort study	To investigate the incidence of BPPV specifically among patients with dizz. in the rehabilitation phase of concussion recovery and to provide evidence regarding the importance of BPPV assessment in physical therapy concussion evaluations	50 (18–85 yrs)	M = 14 F = 36	Concuss^	NR	✓ (20)	✓ (12)		✓ (1)	✓ (17)	Mean 32.68 dys	No cognitive impairment, severe arthritis, radiculopathies, or systemic conditions
Galea et al. (2022) (63)	Australia	Observational cohort study	(1) to identify whether adults 4 wks to 6 mths post mTBI have sensorimotor impairments compared with controls without mTBI. (2) to determine if sensorimotor impairments were evident irrespective of participant perceived absence of symptoms	74 mTBI 39 Control^6^ (18–60 yrs)	M = 60 F = 53	Mild	NR	✓ (17)	✓ (10)	✓ (9)	✓ (38)		Avg. 72 dys	No neurological, psychiatric, visual, or vestibular impairments. No substance abuse or intracranial bleed
Brown et al. (2022) (75)	Australia	An exploratory study	(1) to compare the results of the VOMS in combat sport athletes with a healthy control population. (2) to explore differences between athletes with and without a concussion history. (3) to examine the relationship between VOMS and the Post-Concussion Symptom Scale in combat sport athletes	40 (18 Concuss^) 40 Control^7^ (Mdn: 26 yrs)	M = 80	Concuss^	Self-defined: Concussion in Sport grp definition (111)				✓		Mean 9.8 ± 9.4 wks	Competitive fight within last 2 yrs
Honaker et al. (2015) (71)	USA	Cross-sectional study	To describe the performance of the Gaze Stabilization Test in a cohort of collegiate football players and to examine the effects of previous concussion on outcome parameters of the Gaze Stabilization Test	15 Concuss^ 25 w/o Concuss^ (18–23 yrs)	M = 80	Concuss^	NR				✓		3 mths–9 yrs	n15: prev. Concuss^. No orthopaedic conditions, no neck/back injuries and no vision impairment at 10 feet
SmullIgan et al. (2024) (74)	USA	Cross-sectional study	To investigate dizz., vestibular/oculomotor symptoms, and cervical spine proprioception among adults w and w/o a concussion history	42 Concuss^ 46 w/o Concuss^ (18–40 yrs)	M = 21 F = 67	Concuss^	NR				✓		6 mths–3 yrs	No neurological conditions and limited physical activity
Lin et al. (2015) (93)	Taiwan	NR	It examined the variations in balance function and sensory integration that occur within 1 wk. following an mTBI and compared the differences with those observed in healthy control pts	107 mTBI (mean: 34.8 ± 14.8 yrs) 107 Control^8^ (mean: 32.9 ± 11.1 yrs)	M = 150 F = 64	Mild	Loss of conscious <30 min. GCS: 13–15 Post-traumatic amnesia <24 h	✓ (33)	✓ (51)	✓ (18)	✓ (3)	✓ (2)	1 wk. Avg. of 3.7 ± 1.2 dys	No history of epilepsy, cerebrovascular disease, mental retardation, neurodegenerative disorders
Campbell et al. (2021) (62)	USA	NR	To identify peripheral vestibular, central integrative, and oculomotor causes for chronic symptoms following mTBI	58 mTBI 61 Control^8^ (18–61 yrs)	M = 40 F = 79	Mild (Chronic)	Loss of conscious <30 min. GCS: 13–15 Post-traumatic amnesia <24 h	✓ (8)	✓ (31)		✓ (7)	✓ (12)	Mean 343 dys	No significant vestibular signs and cerebrovascular disease. No mod. to severe substance abuse
Calzolari et al. (2021) (67)	UK	NR	It was investigated the brain mechanisms of imbalance in acute TBI, its link with vestibular agnosia, and potential clinical impact	37 TBI 35 Matched^8^ (18–65 yrs)	M = 42 F = 32	Mild (n4) Mod-severe (n33)	Mayo TBI severity classification system (112)	✓ (20)	✓ (14)	✓ (3)			2–77 dys	No active pre-morbid medical, neurological or psychiatric condition, musculoskeletal condition impairing ability to balance, substance abuse history
Felipe and Shelton (2020) (82)	USA	NR	To evaluate subclinical cervical abnormalities in the vestibulospinal pathway in pts. w/ a concussion history, w/ and w/o related symptoms, using c-VEMP	45 Normal^9^ 45 Control^10^ 33 History^11^ 27 Symptom^12^ (19–24 yrs)	*F* = 154	Concuss^	NR				✓		NR	No significant audio-vestibular signs and cerebrovascular disease
Christy et al. (2019) (83)	USA	NR	Compare athletes with and without sport-related concussions on the subtests	28 Concuss^ 87 Control^13^ (18–24 yrs)	M = 65 F = 50	Concuss^	NR				✓		17 pts.: 72 h 11 pts.: within 2 wks	n13: Prev. concuss^
Gard et al. (2022) (72)	Sweden	NR	To establish the cause of vestibular impairment in athletes with concussion who have persisting post-concussive symptoms	21 w/ prev. Concuss^ 21 Control^13^ (18–43 yrs)	M = 25 F = 17	Concuss^	NR				✓		Min. 6 mths ff concuss^	No prev. or current self-reported neurological or psychiatric disorder A history of at least one sports related concuss^
Kim et al. (2024) (66)	Korea	NR	To evaluate the characteristics of head trauma and brain injury and assess the relationship between them and treatment outcomes in patients with t-BPPV	63 (18–62 yrs)	M = 34 F = 29	NR	NR	✓ (34)	✓ (25)	✓ (4)			2–15 dys	No history of other labytrinthine or central nervous system disorders No prev. Vertigo

^*^Sample size in case series larger.

a Sample sizes varies across tests.

1 BPPV group: those with TBI and posterior canal BPPV.

2 NSD group: those with TBI and nonspecific dizziness.

3 No dizz: those with TBI and no dizziness.

4 Symptomatic: “feeling dizzy” and “loss of balance” on the Neurobehavioural Symptom Inventory.

5 Asymptomatic: No report of “dizziness” and “loss of balance” on the Neurobehavioural Symptom Inventory.

6 No prior concussion history.

7 Healthy controls >15 min of physical activity at least 3 times per week.

8 Healthy controls matched for age and sex with TBI patients.

9 Without neurologic complaints and with a normal clinical examination result.

10 Athletes with no history of concussion.

11 Athletes with concussion history but no symptoms.

12 Athletes with a definite concussion and symptoms.

13 Healthy athletes.

Avg., Average; C, Case; Concuss^, Concussion; dizz., dizziness; dys, days; F, Female; ff, following; f/u, follow-up; hrs, hours; GCS, Glasgow Coma Scale; grp, Group; Prev., Previous; M, Male; min, minimum; MVA, Motor Vehicle Accidents; mdn, Median; mTBI, mild TBI; mths, months; mod, moderate; NR, Not reported; NSD, nonspecific dizziness; Prev., Previous; Pts, Participants; SNHL, Sensorineural hearing loss; SRC, Sports-related concussion; wks, weeks; w/, with; w/o, without; yrs, years.

### 3.2 Participant characteristics

Across 50 records, 1,793 participants were included. Of these, 1,218 were in the patient group (all group participants who had TBI, whether they had symptoms or not), whilst 575 were in the control group (either healthy controls or with vestibular symptoms). Pre-TBI status or history of participants are shown in Table 1, however this information was not consistently reported across all studies. Assessment time since injury varied widely across studies, ranging from as early as 2 days (24) to an average of 584.5 days (51) (Table 1). Four sports-related concussions studies included a pre-session baseline assessment (52–55). In 8 studies, follow-up/s’ assessments were performed after the initial time of injury before any treatment was offered (24, 25, 28, 31, 34, 38, 40, 49).

### 3.3 Overview of vestibular assessments and impairments following non-blast related TBI

Many different symptoms were reported in included studies, from brief dizziness experienced with changes in head position to prolonged dizziness and vertigo accompanied by nausea. These symptoms were assessed using a variety of tests and patient-reported outcome measurements (PROMs). The following sections briefly describe the vestibular assessments, PROMs and findings. A detailed summary of the assessments and PROMs is presented in Supplementary Appendix Table 3, and the results are demonstrated in Table 2. The vestibular impairments reported in the records are presented in Supplementary Appendix Table 4.

**Table 2:** Vestibular findings of included studies.

Ref	Gender	Severity of TBI	Fall	MVA	Assault/ direct impact	Sports injury	Other	Patients reported auditory symptoms	Vestibular	PROMS
Schuknecht and Davison (1956) (36)	M	NR		✓				C3: Severe vertigo, nausea for dys 3–4 wks: vertigo ↓, but rightward sway	**Caloric test (ice water):** RE no response, LE normal	
Preber and Silversklöld (1957) (38)	M = 2 F = 2 C1: M C2: F C3: M C5: F	NR	✓ (1) C5	✓ (3) C1 C2 C3				C1/2: Vertigo w/ position changes C3: Dizz. w/ position changes, rotational changes (1/2 min.) C5: Vertigo, unconscious	**C1:** 1 mth: **Nylen’s method/Cawthorne’s postural test:** Horizontal Ny. to left in supine hanging position **Rotatory tests:** 60°/s rotatory impulse **Calorigram/Cupulogram:** Central vestibular tonus difference, directional preponderance left 2 mths: **Postural tests:** Negative **Cupulogram:** Normal 4 mths: **Nylen’s method:** Long duration Ny **Cawthorne’s postural test:** Paroxysmal positional vertigo (very slight Ny. to left) **C2:** 3 mths: **Nylen’s method:** Short duration vertical Ny. in the supine hanging position 3 yrs.: **Nylen’s method:** No spontaneous Ny **Cawthorne’s postural test:** Vertical Ny. in the direction of the forehead **Calorigram/Cupulogram:** Normal Paroxysmal positional vertigo **C3:** 3 mths: **Cawthorne’s postural test:** Short duration paroxysmal vertigo; directional preponderance right **ENG:** Spontaneous Horizontal right, eyes closed **Cupulogram:** Directional preponderance right **C5:** 2 mths: **ENG:** No Ny. in any position **Caloric test:** Normal 1 yr.: **Cawthorne’s postural test:** Negative; **ENG**: Ny. To left, eyes closed **Calorigram/ Cupulogram:** Directional preponderance left 1.2 yrs.: **Nylen’s method:** No spontaneous Ny **Cawthorne’s postural test:** Ny. to left Paroxysmal positional vertigo	
Tonkin and Fagan (1975) (44)	M = 3 C7 C9 C10	NR	✓ (3)					C7: Unsteady on feet C9: Constant imbalance & unsteadiness C10: Nausea & rotatory vertigo	**C7:** **ENG:** Right-positioned, direction-fixed spontaneous Ny **Caloric test:** Left caloric hypofunction of 68.9% **C9:** 8 mths: **ENG:** Left spontaneous, direction & position-fixed Ny.; left vestibular hypoactivity 55% **C10:** **ENG:** Left spontaneous Ny **Caloric test:** Normal	
Herdman (1990) (49)	M = 2 F = 1 C1: M C3: M C2: F	NR	✓ (2) C1 C2	✓ (1) C3				C1: Dizz., vomiting, headache, blurred vision, diplopia & ataxia C2: Loss of consciousness, nausea, vertigo w/ position changes & vomiting C3: oscillopsia & gait ataxia	**C1:** 5 dys: **ENG:** Mild gaze-evoked Ny., decreased function of right labyrinth Diagnosis: post-concussion syndrome & labyrinthitis 2 yrs.: **Rotational testing:** Normal gain but consistent w/ a unilateral peripheral lesion **Saccades:** Hypometric & slow **Pursuit:** Poor, especially on right **Optokinetic Ny**.: Decreased **Caloric test:** Severely hypoactive left labyrinth **Dynamic Posturography:** Normal Anterior/posterior sway w/ eyes open on stable surface; Abnormal increased sway w/ eyes closed **C2:** 10 wks: **Head Impulse Test:** Normal, no corrective saccades to rapid head movements **Pursuit:** Normal **Saccades:** Normal, no corrective saccades to rapid head movements **VOR/ VOR cancellation:** Normal; No Spontaneous Ny; No Gaze-evoked Ny **Head-Shaking Test**: No Head Shaking induced Ny **Dix-Hallpike Manoeuvre:** Torsional Ny. w/ fast phases counterclockwise, when eyes directed left, RE **C3:** 12 dys: **Caloric test**: B/L no response to cold/ warm water irrigation 4 mths: **Rotational testing:** B/L severe vestibular deficit **Dynamic Posturography:** Unable to maintain balance w/ distorted or absent visual & proprioceptive cues	
Lyos et al. (1995) (42)	M = 3	NR	✓ (1) C2		✓ (1) C3		✓ (1) C1	C1: Nausea, vertigo C2: Imbalance C3: Severe vertigo, headaches, nausea & vomiting	**C1:** **Neurotologic:** Spontaneous right-beating Ny **Fistula test:** Left Negative **C2:** **ENG (Caloric test):** B/L no response **C3:** **ENG (Caloric test):** RE no response	
Kleffelgaard et al. (2016) (46)	M = 2 F = 2 C1: M C2: M C3: F C4: F	Mild	✓ (2) C3 C4	✓ (1) C1	✓ (1) C2				**C1:** **mCTSIB:** Normal **Oculomotor tests:** Normal but symptomatic, eye strain, nausea, forehead pressure **Head Thrust test/DVAT/Dix-Hallpike Manoeuvre/Roll Test:** Negative **C2:** **mCTSIB:** Increased sway eyes closed on the foam surface **Oculomotor tests:** Normal but symptomatic, eye strain **Head Thrust test/ Dix-Hallpike Manoeuvre/ Roll Test**: Negative **DVAT:** Positive (≥ 4 lines difference) **C3:** **mCTSIB:** Increased sway backward & to right, eyes closed on firm/foam surface **Oculomotor tests:** Normal but symptomatic, eye strain in right eye, dizzy **Head Thrust test/DVAT**: Negative **Dix-Hallpike Manoeuvre:** Positive right posterior SCC **Roll Test:** Positive horizontal SCC **C4:** **mCTSIB:** Increased sway, eyes closed on firm/foam surface **Oculomotor tests:** Normal but symptomatic, eye strain, dizzy **Head Thrust test/DVAT/Dix-Hallpike Manoeuvre/Roll Test:** Negative	C1: **DHI:**48 (mod impair.) **VSS-SF:** 19 C2: **DHI:** 56 (severe impair.) **VSS-SF:** 19 C3: **DHI:** 74 (severe impair.) **VSS-SF:** 42 C4: **DHI:** 48 (mod impair.) **VSS-SF:** 17
Feneley and Murthy (1994) (24)	M	NR	✓					Support to sit upright; fell to sides. Remained unsteady standing w/ support	3 dys: **Caloric test (bithermal air):** B/L no response (indicating B/L canal paresis) > 6 mths: **Caloric test:** B/L labyrinthine hypofunction; minimal responses from both vestibules	
Bertholon et al. (2005) (25)	M C1	NR	✓ C1					Positional vertigo when rolling onto right or left side in bed	14 dys: **Dix-Hallpike Manoeuvre:** Right posterior SCC BPPV **Horizontal Canal Manoeuvre:** An ageotropic horizontal SCC, possibly the right horizontal SCC 18 dys: **Horizontal Canal Manoeuvre:** Persistence ageotropic horizontal Ny. 1 mth: **Horizontal Canal Manoeuvre:** Spontaneously disappeared	
Lerut et al. (2007) (40)	F	NR	✓					Vertigo	5 dys: **Dix-Hallpike Manoeuvre:** B/L posterior BPPV 2 mths: **Dix-Hallpike Manoeuvre:** B/L negative	
Fujimoto et al. (2007) (31)	M	NR	✓					NR	1 mth: **Caloric test (ice water stimuli):** B/L normal responses 23 mths: **Caloric test:** 31% canal paresis on right side **VEMP:** B/L **n**ormal	
Johnson (2009) (50)	F	Mild	✓					Chronic vertigo; initial vertigo, nausea & emesis. Intermittent position-provoked vertigo (<1 min) w/ nausea; no recent emesis	**Dix-Hallpike Manoeuvre:** Negative on the left, right torsional up-beat Ny. on the right Right posterior SCC canalithiasis BPPV	
Ralli et al. (2010) (30)	M C1	NR	✓ C1					Shortly severe vertigo	**Dix-Hallpike Manoeuvre:** Right posterior SCC BPPV	
Dlugaiczyk et al. (2011) (60)	M C2	NR	✓ C2					Strong vertigo in bed, bending over, or looking up	**Dix-Hallpike Manoeuvre:** Right anterior & posterior SCCs BPPV	
Chung et al. (2011) (48)	M	NR	✓					Mild dizz. & ataxia	**Caloric test:** B/L no response	
Sousa Menezes et al. (2019) (39)	M	NR	✓					Severe dizz., vertigo, otalgia or otorrhea	**Spontaneous & Gaze Ny.:** Mixed horizontal-torsional grade II right beating Ny **Hennebert Sign:** Positive Ny. on the left	
Jafarzadeh et al. (2022) (65)	M = 20 F = 1	Mild	✓					n21: persistent vertigo or n14: imbalance	**Bedside examination:** Abnormal results in Gaze (19%, n4), Abnormal results in Pursuit tests (38%, n8), Normal spontaneous Ny, Normal saccade **Dix-Hallpike Manoeuvre, Side-lying Manoeuvre, Roll test:** Posterior SCC BPPV in 6 pts. (28.5%), B/L BPPV (n1) **c-VEMP:** Abnormal saccular function (66.6%, n14), 6 out of 14 had B/L saccular abnormality **SOT:** No significant difference between pts. w/ normal & abnormal saccular function	**DHI:** Total: 37 ± 24.9 No significant difference between pts. w/ normal & abnormal saccular function
Ylikoski et al. (1982) (27)	M = 2 C8 C9	NR		✓ (2)				C8: Constant unsteadiness; unable to walk straight C9: Constant unsteadiness w/ occasional vertigo attacks	**C8:** **Caloric test:** No response in the right **C9:** **Caloric test:** 75% right reduced vestibular response	
Jani et al. (1991) (35)	F	Mod-Severe		✓				NR	**Caloric test (ice water):** B/L normal Ny.	
Fitzgerald (1995) (29)	F C1	NR		✓				Positional vertigo, motion intolerance	**C1:** **Fistula test**: Negative in subjective, right positive in platform 10 wks: **ENG:** Right reduced vestibular response	
Ottaviano et al. (2009) (32)	F C2	NR		✓ C2				Vertigo	**C2:** **VNG:** Left post-traumatic Benign positional vertigo	
Kagoya et al. (2010) (26)	F	NR		✓				Dizz. w/ head position changes; subsided over 2 M	**Fistula test:** No fistula sign **Caloric test:** Normal	
Paxman et al. (2018) (43)	M	Mild		✓				Persistent dizz., light-headed, disorientation, nausea, fatigue. Worse w/ activity, posture/ contrast changes, convergence, improved w/ rest	**VEMP/ VNG:** Normal **Computerised dynamic posturography:** Normal **Dix-Hallpike Manoeuvre/ Vision assessment:** NR **Vision assessment (visual acuity, eye tracking, visual fields and convergence)**: NR in detail All investigations suggested normal peripheral & central vestibular functioning	
Roup et al. (2020) (28)	F	Mild		✓				Dizz. & problems w/ balance	8 mths: **Neuro-vision evaluation: S**ignificant functional vision deficits/visual-vestibular dysfunction. Diagnosis: convergence insufficiency, pursuit eye movement deficit 23 mths: **VNG:** Normal	
Waninger et al. (2014) (37)	M	Concuss^				✓		NR	**Neurological examination:** Abnormalities on vestibular testing, eye accommodation & convergence	
Honaker et al. (2015) (71)	M = 80	Concuss^				✓		Athletes w/ previous concuss^: 13% = 2 concuss^, 13% = 3 concuss^, 6% = 4 concuss^	**Oculomotor Screening:** Abnormalities for both grps but no statistically significant differences w/ Previous concuss^ grp: **-*Smooth pursuit:*** 13% (n2) pts. had impairment ***-Saccades:*** 20% (n3) pts. had impairment ***-Gaze stability w/ fixation:*** 7% (n1) pts. had impairment ***-Gaze stability w/o fixation:*** 33% (n5) pts. had impairment **VOR Function Screening:** Abnormalities for both grps but no statistically significant differences. w/ Previous concuss^ grp: ***-Horizontal head thrust:*** 13% (n2) pts. had impairment ***-Horizontal head shaking:*** 20% (n3) pts. had impairment **Gaze Stabilization Test:** significantly larger GST asymmetry score Diagnosis: Peripheral vestibular or vestibular-visual interaction deficits	**DHI:** No significant differences between grps. The range of DHI scores for athletes w/ previous concuss^ was much wider than the comparison grp Mean in previous concuss^ grp: 3.60
Hides et al. (2017) (52)	NR	Concuss^				✓		NR	**Vestibular system:** No significant differences pre/post-concuss^ **Oculomotor assessment:** NR **VOR gain:** Outside the normal range for 2 pts. **vHIT:** Increased asymmetry for 3 pts. **Dix Hallpike Manoeuvre, Head Roll:** No BPPV pre & post-concuss^	**DHI:** Mean in post-concuss^: 2.6 (5.3)
Christy et al. (2019) (83)	M = 65 F = 50	Concuss^				✓		NR	**Rotary Chair:** No difference in VOR gain or phase between pts. w/ concuss^ or w/o concuss^; Significantly worse scores (*p* < 0.05) in VOR cancellation gain **c-VEMP:** No significant differences between pts. w/ concuss^ or w/o concuss^ **SOT:** Significantly worse scores all conditions in pts. w/ concuss^ Peripheral vestibular system & brainstem/cerebellar VOR pathways was unaffected. Diagnosis: Impairment of central integration of vestibular function	
Felipe and Shelton (2020) (82)	F = 154	Concuss^				✓		Symptom grp: dizz., persisting > 10 dys	**c-VEMP:** The symptom grp: Significantly higher latency scores than the control & normative grps in P13 & in N23. 32.3% abnormal responses. The history grp: Statistically higher latency scores than the control & normative grps 24% abnormal responses. Possible diagnosis: Impairment in saccular or vestibulocollic function	
Glendon et al. (2021) (54)	M = 25 F = 17	Concuss^				✓		NR	2 dys: **VOMS:** 57.1% (n24) pts. had impairment & VOMS score increased from baseline at 2D post-concussion. ***-Smooth pursuit:*** 38.1% (n16) had impairment ***-Horizontal saccades:*** 40.5% (n17) had impairment ***-Vertical saccades:*** 42.9% (n18) had impairment ***-Horizontal vestibular-ocular reflex:*** 47.6% (n20) had impairment ***-Vertical vestibular-ocular reflex:*** 35.7% (n15) had impairment ***-Visual motion sensitivity test:*** 28.6% (n12) had impairment ***-Near point convergence:*** 17.7% (n7) had impairment. Diagnosis: Vestibulo-oculomotor dysfunction	**PCSS**: PCSS symptom scores from pre-session baseline were significantly greater in those with impairment on VOMS at all time points except return-to play
Teramoto et al. (2022) (53)	M = 59 F = 52	Concuss^				✓		NR	**VOMS:** ***-Smooth pursuit/ Horizontal saccades/Vertical saccades:*** Increase in post-concuss^ symptom scores compared to pre-concuss^ ***-Convergence/ Horizontal vestibular-ocular reflex/ Vertical vestibular-ocular reflex:*** No significant change	
									**Effect of gender:** M/F pts. had significantly higher scores at post-injury than pre-injury F reported more symptoms than M in all categories but there were statistically significant differences in smooth pursuit, horizontal saccades & vertical saccades	
Brown et al. (2022) (75)	Athlete: M = 40 Control: M = 40	Concuss^				✓		Self-reported history of concuss^	**VOMS:** No significant differences between grps w/ & w/o a history of concuss^ 38.9% (n7) pts. w/ a concuss^ history scored outside the clinical cutoff on at least one of subtests Abnormal NPC distance in 44.4% of pts. w/ history of concuss^	
Gard et al. (2022) (72)	SRC: M = 14 F = 7 Control: M = 11 F = 10	Concuss^				✓		Vestibular disturbance	Vestibular dysfunction in 13 of 21 pts. w/ SRC (Peripheral: 9, central & peripheral: 4) **vHIT:** 52% (n10) of pts. w/SRC had abnormal results **Caloric test:** 24% (n5) of pts. w/SRC had abnormal results **c-VEMP:** 38% (n8) of pts. w/SRC had abnormal results **VNG:** 14% (n3) of pts. w/SRC had abnormal results **Posturography:** 38% (n8) of pts. w/SRC had abnormal results **Pursuit eye movements**: 19% (n38) of pts. w/SRC had abnormal results	**DHI:** Higher scores pts. w/ SRC compared w/ controls When vestibular pathology existed, pts. scored higher on DHI (mdn 35, IQR 4.5-47)
									**Effect of gender:** Vestibular dysfunction did not correlate with gender	**Effect of gender:** No correlation with DHI
Smulligan et al. (2024) (74)	M = 21 F = 67	Concuss^				✓		NR	**Visio-vestibular exam:** Significantly more positive vestibular/ocular subtests in the concuss^ history grp compared to those w/o concuss^ history grp Dizz. & vestibular/ocular symptoms were associated among the concuss^ grp Diagnosis: Persist chronic vestibulo-oculomotor symptom provocation	**DHI:** More severe dizz. Symptoms in concuss^ history grp
									**Effect of gender:** There is no statistically significant association between gender & performance on the Visio-vestibular exam	**Effect of gender:** No statistically significant association between gender and DHI
Uyeno et al. (2024) (55)	M = 63 F = 56	Mild				✓		NR	**Eye-tracking assessment:** 44% of pts. had abnormal eye-tracking post-mTBI; No significant differences were observed between baseline & post-TBI scores on the eye-tracking metrics *Repeat injury:* no significant change eye-tracking proficiency compared w/ baseline or increase the frequency of abnormal eye-tracking scores **VOMS:** Horizontal gain had med-large positive correlation w/ headache (r0.34) & dizz. (r0.54) Diagnosis: Central or peripheral vestibulopathy	
Kanavati et al. (2016) (33)	M	NR			✓			Dizz.	**Head impulse test:** Normal	
Durbec et al. (2012) (41)	M	NR			✓			Loss of balance	**VNG w/ caloric test:** Total right vestibular areflexia	
Jacobs et al. (1979) (47)	F C1	NR					✓	Intermittent nausea, light-headedness, & vertigo	**C1: ENG (Caloric test):** Symmetrical, No spontaneous Ny.	
Mohd Khairi et al. (2009) (45)	M	NR					✓	NR	**Caloric test:** Right canal paresis	
Blackard et al. (2020) (34)	F	Concuss^					✓	Mild concuss^ symptoms	5 dys: **VOMS: *Horizontal pursuit test:*** Ny. in left eye upon rightward eye movement **-*Vertical pursuit test:*** The right eye displays a noticeable lag **-*Horizontal saccades test*:** Negative -***Vertical saccades test:*** Positive increased dizz. ***-Convergence test:*** Normal convergence ranging from 4-6 cm. The right eye did not converge during testing. 1 mth: Abnormal findings in vestibular function but NR in detail. Diagnosis: Vestibulo-oculomotor dysfunction	
Lin et al. (2015) (93)	mTBI M = 75 F = 32 Control: M = 75 F = 32	Mild	✓ (33)	✓ (51)	✓ (18)	✓ (3)	✓ (2)	NR	**mCTSIB:** Sensory integration dysfunction in mTBI grp. Significant differences existed between mTBI & control grps in eyes open whilst standing on a firm surface. Diagnosis: Sensory integration dysfunction	**DHI:** pts. w/ mTBIs significantly increased scores compared w/ control. Mean score in mTBI: 26 (21.9)
Campbell et al. (2021) (62)	Chronic mTBI: M = 16 F = 42 Control: M = 24 F = 37	Mild	✓ (8)	✓ (31)		✓ (7)	✓ (12)	> 3 mths post mTBI w/ non-resolving balance complaints (for mTBI grp)	**Peripheral vestibular assessment (vHIT; c-VEMP; o-VEMP; Bithermal caloric test):** Normal in the chronic mTBI compared to the control; no significant differences. Largest percentage caloric unilateral weakness in pts. w/ abnormal function within the chronic mTBI grp. **Oculomotor assessment (Horizontal random saccades; Horizontal smooth pursuits):** Normal in chronic mTBI compared to the control; no significant differences. Largest percentage smooth pursuit velocity gain in pts. w/ abnormal function within the chronic mTBI grp. **Dix-Hallpike Manoeuvre:** Positive in only one pt. **SOT:** Significantly higher proportions of abnormal responses in the chronic mTBI grp compared to the control grp Diagnosis: Central sensory integration dysfunction	**NSI:** Worse NSI affective subscore mod significantly correlated w/ lower vHIT-VOR gains. Vestibular subscore significantly correlated w/ worse performance on the SOT **DHI:** Physical subscore correlated w/ worse performance on the SOT
Galea et al. (2022) (63)	mTBI: M = 43 F = 31 Control M = 17 F = 22	Mild	✓ (17)	✓ (10)	✓ (9)	✓ (38)		NR	**Oculomotor assessment-VNG (SKED, OKN)/ Video vestibulo-ocular reflex test (VVOR, VORS):** More positive in the mTBI grp compared to control. OKN & VORS were more positive for both SYMP & ASYMP subgroups ***vHIT:*** Positive in 14.9% (n10) of mTBI grp **Vestibular positional tests:** ***-Dix-Hallpike Manoeuvre:*** Positive in 20.9% (n14) in mTBI grp ***-Head roll:*** Positive in 19.4% (n13) in mTBI grp Diagnosis: Persistent sensorimotor impairment	**DHI-Short form:** mTBI grp (mdn 12(4)) & SYMP had greater DHI scores than controls & ASYMP grp **SMD II:** mTBI (mdn 2.22 (6.4) & SYMP grp had higher scores than the controls & the ASYMP grp
Ouchterlony et al. (2016) (61)	M = 34 F = 22	Mild Mod.	✓ (23)	✓ (23)	✓ (3)	✓ (5)	✓ (2)	BPPV: 90.5%; NSD: 76.2% spinning w/ dizz. Both: Light-headedness, dizz. Affected by position	**Dix-Hallpike manoeuvre:** 21 pts. (both in BPPV & NSD grp) had BPPV (B/L BPPV n4 (2.8%); U/L BPPV n16 (76.15%)	**DHI:** Pts in both BPPV (mean: 42.9) & NSD grps (mean: 51) showed high levels of impairment at pre-session baseline
									**Effect of severity** BPPV grp had significantly more in pts. with mod. TBI (23.8%) than the NSD grp All of NSD grp had mild TBI	
									**Effect of aetiology (BPPV)** **Fall:** 11 pts.; MVA: 8 pts.; Sports injury: 2pts **Assault**: No BPPV	
									**Effect of gender:** **M:**15, **F:**6 had BPPV	
Calzolari et al. (2021) (67)	TBI M = 26 F = 11	Mild Mod-Severe	✓ (20)	✓ (14)	✓ (3)			NR	**vHIT:** No significant vestibular deficit **Caloric test:** Only 2 pts. tested & were normal **Rotational chair:** The gain was within normal limits **BPPV screening**: Of these 37 pts., 18 had BPPV **VOR thresholds assessment:** Elevated VOR thresholds in acute TBI **Vestibular-motion perceptual thresholds:** Dramatically elevated compared to controls; 15 of 37 pts. w/ acute TBI had vestibular agnosia **Static Posturography:** TBI pts. w/ vestibular agnosia were more unstable than controls in all conditions; TBI pts. w/o vestibular agnosia were more unstable than controls in vestibular-mediated condition (soft surface w/ eyes closed); TBI pts. w/ vestibular agnosia were more unstable than pts. w/o vestibular agnosia on both soft surface conditions	**DHI:** Acute TBI pts. w/ vestibular agnosia (22.5 ± 17.1) & w/o vestibular agnosia (29.7 ± 22.5) reported mod. Dizz. symptoms No differences between TBI pts. w/ & w/o vestibular agnosia
									**Effect of severity: BPPV screening (n18 BPPV):** 15 pts. with mod-severe TBI 3 pts. with mild TBI	
									**Effect of aetiology: BPPV screening:** **Fall**: 13 pts., **MVA**: 5 pts	
									**Effect of gender: BPPV screening:** **M**: 14 pts., **F:** 4 pts	
Taylor et al. (2022) (68)	M = 40 F = 59	Mild Mod	✓ (36)	✓ (17)	✓ (5)	✓ (19)	✓ (22)	Dizz. and/or balance symptoms	33 pts. (33.3%) had abnormalities involving one or more vestibular organs/afferent divisions **vHIT:** n16 had abnormal results, (horizontal SCC, n7; posterior SCC, n7; anterior SCC n2); 3 pts. had B/L abnormalities **c-VEMP:** n14 had abnormal results; **o-VEMP:** n8 had abnormal results. **VNG:** Abnormalities on one or more tests of central oculomotor function in 39 out of 95 pts. ***-Gaze-evoked Ny.:*** Bidirectional gaze-evoked or vertical Ny. in darkness in 6%; ***-Horizontal saccades:*** Prolonged latencies in 18/9%, slower velocities in 12.6%, inaccurate saccades in 3.2% -***Smooth Pursuit:*** Abnormalities in 23.4% *-**VOR suppression:*** Poor VOR suppression in 3.6% **Positional Tests:** n9 had BPPV **Caloric test:** n14 had abnormal results **SOT**: 71 out of 94 pts. (75.5%) had abnormal results on one or more SOT scores. A significant relationship between the presence of central oculomotor dysfunction and abnormal postural sway composite SOT scores. Pts w/ oculomotor dysfunction had greater difficulty using vestibular input for balance	
									**Effect of aetiology:** **Fall:** 9 pts. abnormal c-VEMP; 3 pts. abnormal o-VEMP; 5 pts. positional tests; 9 pts. abnormal caloric test; 13 pts. Abnormal vestibular SOT component. Falls as the cause of TBI were common in the grp with vestibular hypofunction. **MVA:** 1 pt. abnormal c-VEMP; 3 pts. abnormal o-VEMP; 1 pt. positional tests; 1 pts. abnormal caloric test; 3 pts. Abnormal vestibular SOT component. **Sport injury:** 1 pt. abnormal c-VEMP; 1 pt. positional tests; 1 pt. abnormal caloric test; 2 pts. Abnormal vestibular SOT component. **Assault:** 1 pt. abnormal o-VEMP; 1 pt. Abnormal vestibular SOT component. **Other:** 3 pt. abnormal c-VEMP; 1 pt. abnormal o-VEMP; 3 pts. abnormal caloric test; 2 pts. Abnormal vestibular SOT component.	
									**Effect of gender:** No significant relationship between presence of abnormalities on vestibular function tests & gender	
McCormick et al. (2023) (64)	M = 14 F = 36	Concuss^	✓ (20)	✓ (12)		✓ (1)	✓ (17)	Dizz.	n11 (22%) had positive BPPV **Dix-Hallpike Manoeuvre:** 8 Posterior SCC BPPV (n7 Canalithiasis & n1 Cupulolithiasis); **Supine Head Roll test:** 3 Horizontal SCC BPPV (Canalithiasis)	**DHI:** M: 39.45 for BBPV grp No significant difference between TBI pts. w/ positive BPPV & w/ negative BPPV
									**Effect of aetiology: BBPV:** **Fall**: 8 pts. **MVA**: 1 pt. **Other**: 2 pts. **Sports injury:** No BPPV Fall was the only statistically significant aetiology of injury; 72.7% of BPPV-positive pts. reported a fall as the aetiology of injury. All BPPV cases related to falls involved the posterior SCCs	
									**Effect of gender:** **M:** 1 pt.: LE horizontal SCC canalolithiasis type BPPV **F:** 5 pts.: LE posterior SCC canalolithiasis type BPPV; 1 pt.: RE posterior SCC canalolithiasis type BPPV; 1 pt.: LE posterior SCC cupulolithiasis type BPPV; 1 pt.: RE horizontal SCC canalolithiasis type BPPV; 1 pt.: LE horizontal SCC canalolithiasis type BPPV; 1 pt.: Bilateral posterior canalolithiasis type BPPV	
Kim et al. (2024) (66)	M = 34 F = 29	NR	✓ (34)	✓ (25)	✓ (4)			NR	**Dix-Hallpike Manoeuvre, Head Roll test:** Single canal involvement BPPV in 52 (83%); Mostly posterior SCC (79%), Horizontal SCC cupulolithiasis (13%); Multiple canal involvement BPPV in 11 (17%); 6 out of 11 had B/L BPPV; Mostly posterior SCC	
									**Effect of gender:** Mostly posterior SCC post-traumatic BPPV was more prevalent in M than in F, which may be attributed to gender differences in the incidence of TBI	
Joseph et al. (2021) (51)	M = 31 F = 19	Mild (n24) Mod (n21) Severe (n5)	✓ (18)		✓ (16)		✓ (16)	NR	**SOT:** No significant differences between grps for any measures. The majority of pts. from both grps scored within the normal range but average scores were worse for the symptomatic grp In the symptomatic grp, 5 pts. had an abnormal vestibular result In the asymptomatic grp, 2 pts. had an abnormal vestibular result	**NSI:** Symptomatic grp (29.1 (19.3)) significantly more neuro-behavioural symptoms than the asymptomatic grp **DHI:** Symptomatic grp (21.4 (20.6)) greater disability (mild impair) than the asymptomatic grp
									**Effect of gender:** No statistically significant relationship between vestibular score & gender in SOT	**Effect of gender:** No significant effect of gender on DHI score in SOT vestibular score.
Motin et al. (2005) (58)	M = 18 F = 2	Severe	✓ (6)	✓ (4)			✓ (10)	Vertigo w/ positional changes/physical exertion, relieved by rest; light-headedness, floating, drunkenness sensations	**Dix-Hallpike Manoeuvre:** n10/20 (50%) diagnosis of posterior SCC BPPV. n4 had B/L BPPV. No horizontal SCC BPPV **Head Thrust:** Positive to right in n1 w/ left BPPV & right peripheral vestibular loss **DVAT:** Drop in visual acuity of more than two lines in one pt. w/ left BPPV & right peripheral vestibular loss **Head shaking:** The optic disc was unstable when ophthalmoscopy was performed during head shaking	
									**Effect of aetiology: BPPV:** **MVA**: 4 pts. **Other accident**: 6 pts	
									**Effect of gender: BPPV:** **F:** 2 pts.; **M:** 8 pts	
Ahn et al. (2011) (59)	M = 18 F = 14	NR	✓ (8)	✓ (20)	✓ (4)			Vertigo	**Dix-Hallpike Manoeuvre:** 24 pts. had posterior SCC BPPV w/ canalolithiasis type **Supine head-turning:** 11 had horizontal SCC BPPV (8 pts. had canalolithiasis, 3 pts. had cupulolithiasis). One pt. had B/L BPPV	
									**Effect of aetiology:** **Fall:** 2 pts.: RE posterior SCC canalolithiasis types BPPV; 3pts: LE posterior SCC canalolithiasis types BPPV; 1 pt.: RE horizontal SCC canalolithiasis type BPPV; 1 pt.: RE posterior SCC canalolithiasis & LE horizontal SCC cupulolithiasis types BPPV; 1 pt.: RE posterior & horizontal SCCs canalolithiasis types BPPV **MVA:** 7 pts.: RE posterior SCC canalolithiasis type BPPV; 6 pts.: LE posterior SCC canalolithiasis type BPPV; 3 pts.: RE horizontal SCC canalolithiasis type BPPV; 1 pt.: RE horizontal SCC cupulolithiasis type BPPV; 2 pts.: LE horizontal SCC canalolithiasis type BPPV; 1 pt.: LE posterior SCC canalolithiasis & RE horizontal SCC cupulolithiasis types BPPV **Assault:** 3 pts.: RE posterior SCC canalolithiasis types BPPV; 1 pt.: LE horizontal SCC canalolithiasis type BPPV	
									**Effect of gender:** **M:** 7 pts.: RE posterior SCC canalolithiasis type BPPV; 6 pts.: LE posterior SCC canalolithiasis type BPPV; 1 pt.: RE horizontal SCC canalolithiasis type BPPV; 1 pt.: RE horizontal SCC cupulolithiasis type BPPV; 1 pt.: LE horizontal SCC canalolithiasis type BPPV; 1 pt.: LE posterior SCC canalolithiasis & RE horizontal SCC cupulolithiasis types BPPV; 1 pt.: RE posterior & horizontal SCCs canalolithiasis types BPPV **F:** 5 pts.: RE posterior SCC canalolithiasis types BPPV; 3 pts.: LE posterior SCC canalolithiasis types BPPV; 3 pts.: RE horizontal SCC canalolithiasis type BPPV; 2 pts.: LE horizontal SCC canalolithiasis type BPPV: 1 pt.: RE posterior SCC canalolithiasis & LE horizontal SCC cupulolithiasis types BPPV	

ASYMP, Asymptomatic; B/L, Bilateral; BPPV, Benign paroxysmal positional vertigo; c-VEMP, Cervical vestibular evoked myogenic potential; Dys, day(s); DHI, Dizziness Handicap Inventory; Dizz., Dizziness; DVAT, Dynamic Visual Acuity Test; ENG, Electronystagmography; Esp, Especially; F, Female; Grp, group; IQR, Interquartile Range; LE, Left ear; M, Male; mCTSIB, modified clinical test of sensory integration and balance; min, minutes; mTBI, Mild traumatic brain injury; mths, month(s); mod., moderate; mdn, Median; NR, Not reported; NPC, Near point converfence; NSD, nonspecific dizziness; NSI, Neurobehavioural Symptom Inventory; Impair., Impairment; Ny, Nystagmus; OKN, Optokinetic nystagmus o-VEMP, Ocular vestibular evoked myogenic potential; PCSS, Post-concussion symptom scale; pt(s), Participant(s); RE, Right ear; SCC, Semicircular canal; SKED, skew eye deviation; SMD II, Space and Motion Discomfort II; SOT, Sensory Organization Test; SRC, Sports related concussion; SYMP, Symptomatic; U/L, Unilateral; vHIT, Video Head Impulse Test; VNG, Videonystagmography; VOR, Vestibulo-ocular reflex; VORS, Vestibulo-ocular reflex suppression; VOMS, Vestibular/Ocular Motor Screening; VSS-SF, Vertigo Symptom Scale Short Form; VVOR, Visual vestibulo-ocular reflex; Wks, week(s); w/, with; w/o, without; Yrs, year(s).

#### 3.3.1 Dynamic positional tests

The Dix-Hallpike manoeuvre (56, 57) and the side-lying manoeuvre are used in the differential diagnosis of both peripheral and central types of positional vertigo and posterior and anterior semicircular canals (SCCs) BPPV. The Roll manoeuvre is used to diagnose horizontal SCC BPPV (57). The Dix-Hallpike manoeuvre was reported in 18 studies (25, 30, 38, 40, 43, 46, 49, 50, 52, 58–66) and the side-lying manoeuvre was used in one study (65), whilst both the Dix-Hallpike and Roll manoeuvres (horizontal SCC manoeuvre/supine head turning) were applied in 8 out of the 18 records (25, 46, 52, 59, 63–66). In one study, participants were prospectively audited for the presence or absence of BPPV without any manoeuvres (67), whilst in another study, participants were retrospectively monitored (68).

Positive results in favour of BPPV were observed in 16 out of 18 records in which the Dix-Hallpike and Roll manoeuvres were applied (25, 30, 38, 40, 46, 49, 50, 58–66). Posterior SCC BPPV was reported in 11 of these studies (25, 30, 40, 46, 50, 58–60, 64–66). Horizontal (lateral) SCC BPPV was observed in 6 records (25, 46, 59, 63, 64, 66). Furthermore, the two audit studies, one prospective and one retrospective, Calzolari et al. (67) and Taylor et al. (68) reported the presence of BPPV in participants. Ottaviano et al. (32) reported left post-traumatic benign positional vertigo without specifying the manoeuvre used.

#### 3.3.2 Oculomotor assessment and/or nystagmography (ENG/VNG)

Oculomotor assessment provides a detailed examination of the neurological pathways associated with oculomotor function (69). The Electronystagmography (ENG) and Videonystagmography (VNG) test battery, which provides information about the function of the peripheral and central vestibular system (70), also includes an oculomotor assessment component.

Oculomotor assessment/screening was performed across 4 studies (46, 52, 62, 71). In the 3 studies, Binocular goggles (71), Frenzel goggles (52), or a light bar (62) were utilised; however, there was no detail of using VNG or ENG. Therefore, results from studies utilising VNG/ENG are reported separately from those without such specifications. In 3 studies, oculomotor assessment results were generally found to be normal or did not show significant differences compared to the control group or pre-concussion conditions (46, 52, 62). In one cross-sectional study, although abnormalities were detected in both athlete groups with and without a previous concussion, there was no significant difference in the distribution of normal and abnormal oculomotor findings between the groups (71). The examinations performed within the scope of each oculomotor assessment are provided in detail in Table 2.

In seven studies, eye assessments were presented under various names (e.g., neurological or bedside examination, neurotologic or neuro-vision assessment) (28, 37, 42, 43, 65) or with specific eye assessments such as spontaneous or gaze-evoked nystagmus (39, 49). Although the results were reported differently in each study, visual-vestibular abnormalities were observed in 5 out of 7 studies (28, 37, 39, 42, 65). However, in 1 out of 5 studies, abnormal results were detected in gaze and pursuit tests, whilst spontaneous nystagmus and saccades were normal (65). In remaining 2 studies, the results were normal (43, 49).

Furthermore, ENG (*n* = 6) (29, 38, 42, 44, 47, 49) and VNG (*n* = 7) (28, 32, 41, 43, 63, 68, 72) were utilised across a total of 13 studies. In four studies, one using VNG (41) and three using ENG (42, 47, 49), only caloric test results were presented, rather than ENG or VNG. Therefore, these findings are reported in the next section. Normal results were obtained across 3 studies (28, 38, 43), whilst abnormal VNG or ENG results were observed in 8 studies (29, 32, 38, 44, 49, 63, 68, 72) in at least one case. Notably, a normal VNG result was obtained 23 months post-TBI; however, the initial neuro-visual assessment conducted approximately 8 months after the TBI, revealed visuo-vestibular dysfunction in a case study (28). Abnormalities varied across studies, including findings such as spontaneous nystagmus, reduced vestibular responses, gaze-evoked nystagmus and positive optokinetic nystagmus indicating both peripheral and central vestibular dysfunctions (Table 2).

In an observational cohort study, visual vestibulo-ocular reflex assessment (VVOR) and vestibulo-ocular reflex suppression (VORS) were performed. Individuals with TBI showed more positive results in both the VVOR and VORS compared to the controls (63). Similarly, poor VOR suppression was observed in 3.6% of patients following TBI in a retrospective clinical case series study (68).

Vestibular-oculomotor screening (VOMS) tests the ability to complete vestibular and ocular-related performances and measures the level of symptoms provocation caused by them (73). It was used in 6 studies (34, 53–55, 74, 75). In all 6 studies, at least one subtest of VOMS showed impairment, abnormal findings or an increase in symptoms following concussion/TBI (34, 53–55, 74, 75).

#### 3.3.3 Caloric test

The caloric test evaluates the horizontal SCCs and by extension the superior vestibular nerve (76). The caloric test was performed in 18 out of 50 studies (24, 26, 27, 31, 35, 36, 38, 41, 42, 44, 45, 47–49, 62, 67, 68, 72). In caloric testing, bilateral (24, 42, 48, 49), unilateral (27, 36, 41, 42, 44, 45, 49) vestibular dysfunction (e.g., canal paresis, reduced vestibular responses, vestibular areflexia) or abnormal results (68, 72) were reported in 11 out of 18 studies. One study noted central vestibular tonus differences (38), and 8 studies noted normal caloric responses (26, 31, 35, 38, 44, 47, 62, 67). However, one of these, unilateral canal paresis was observed in the follow-up assessment performed 23 months after the injury (31).

#### 3.3.4 Head thrust/head impulse/video head impulse (vHIT) test

The above tests measure VOR and provides physiological information relating to SCC function (77, 78). Head thrust/impulse (*n* = 5) (33, 46, 49, 58, 71) and vHIT (*n* = 6) (52, 62, 63, 67, 68, 72) were performed in a total of 11 records. Normal (negative) results were obtained in 5 studies (33, 46, 49, 62, 67), whilst findings such as positive, increased asymmetry, and impairment were observed across 6 studies (52, 58, 63, 68, 71, 72). In two studies, the horizontal SCC was assessed (62, 71), while in two studies, all SCCs were reported to be evaluated (68, 72). In seven studies, there was no indication of which SCCs were assessed (33, 46, 49, 52, 58, 63, 67).

#### 3.3.5 Head shaking test

Head-shaking test enables the determination of vestibular asymmetry by rapid head shaking and abrupt stopping movements (79). Head shaking test was used in three records (49, 58, 71). Abnormal results were observed in 2 studies (e.g., unstable optic disc or impairment in previous concussion group) (58, 71), whilst no nystagmus was observed in one study (49).

#### 3.3.6 Vestibular evoked myogenic potential (VEMP) test

Among VEMPs, cervical VEMP (c-VEMP) evaluates the saccule via the sternocleidomastoid muscle, whilst ocular VEMP (o-VEMP) evaluates the utricle via the inferior oblique muscle (80, 81). VEMP measurement was performed in 8 studies (31, 43, 62, 65, 68, 72, 82, 83). In two studies, both c-VEMP and o-VEMP were applied (62, 68), in 4 studies only c-VEMP was performed (65, 72, 82, 83), and in 2 studies, it was not stated which VEMP method was used (31, 43). VEMP results were normal (31, 43, 62) or showed no significant difference between groups with or without TBI/concussion (62, 83) in four studies. In one study, VEMP was performed only at follow-up assessment (31).

In 4 studies, abnormal findings were observed (65, 68, 72, 82). In the group comparison performed by Felipe and Shelton (82), P13 and N23 latency scores were higher in the concussion group with symptoms and asymptomatic concussion groups than in the control and normative groups. However, there was no difference between the two concussive groups in terms of latency scores. Furthermore, only one study reported that bilateral abnormalities were observed (65).

#### 3.3.7 Rotational testing

Rotational/Rotary chair test allows for the assessment of the VOR through the horizontal SCC (84). Rotary chair testing was utilised in 3 studies (49, 67, 83). Another study published in 1957 (38) used cupulometria evaluation (now not widely used), which is similar to the rotational test, and assesses vestibular responses through rotary chair but uses different stimulus magnitudes and durations (85). The results varied depending on the patients or cases, from normal gain to a severe bilateral vestibular deficit (Table 2). Furthermore, in a study comparing healthy athletes to those with concussions, there was no difference in VOR gain or phase (83). However, the timing of the rotary chair test implementation differs across studies (whether during the initial or follow-up assessments).

Furthermore, VOR thresholds and vestibular motion perceptual thresholds were evaluated using a rotary chair in one study (67). Vestibular motion perceptual thresholds measure the smallest appreciable stimulus or perceiving motion (86). In this assessment, dramatically elevated perceptual thresholds were observed in acute TBI patients with vestibular agnosia compared to controls. The VOR threshold was defined as the lowest acceleration required to elicit appropriately directed both slow and fast phases of vestibular nystagmus (87). Elevated VOR thresholds were observed in acute TBI (67).

#### 3.3.8 Dynamic visual acuity (DVA) test and gaze stabilization test (GST)

Dynamic visual acuity (DVA) measures the ability to maintain visual clarity and focus on a target while the head is in motion, reflecting the function of the VOR (88). DVA test was performed in 2 studies (46, 58). A drop in visual acuity was observed in a participant with BPPV on one side and peripheral vestibular loss on the other side (58), whilst there are cases where positive DVA (≥4 lines difference) indicating reduced VOR or negative DVA results were reported following TBI (46).

Another test that evaluates VOR is the GST, which determines the head velocity that causes significant deterioration in visual acuity (89). In one study in which GST was applied, individuals with a previous concussion were found to have significantly larger GST asymmetry scores (71).

#### 3.3.9 Posturography

Posturography provides information on balance function, interactions and impact of sensory system such as visual, vestibular and proprioceptive systems by evaluating body sway through the measurement of the centre of pressure displacement on a force-measuring platform (90). Posturography is divided into two: static and dynamic. Static posturography evaluates changes in the centre of pressure on a fixed platform (91), whilst dynamic posturography measures postural reactions on a moving platform (92). Posturography was used in 11 out of 50 studies (43, 46, 49, 51, 62, 65, 67, 68, 72, 83, 93). Three studies used static posturography (46, 67, 93) and seven studies used dynamic posturography (43, 49, 51, 62, 65, 68, 83). However, it was not specified in one study which type of posturography was used and observed abnormal results in 38% of participants with concussion (72).

The modified clinical test of sensory integration and balance (mCTSIB), one of the subtests of static posturography, was used in 2 studies (46, 93). Although the results were reported differently, unstability, increased sway or sensory integration dysfunction was observed after TBI in static posturography evaluation in all three studies (46, 67, 93). However, there is also a case where normal results were obtained (46). In addition, acute TBI patients with vestibular agnosia were more unstable on static posturography compared to those without vestibular agnosia (67).

Sensory organization test (SOT), which is one of the subtests of dynamic posturography, was performed in 6 studies (49, 51, 62, 65, 68, 83). In one of these studies, it was not stated that SOT was applied, but it was shown in the figure (49). The results were generally reported differently in each record based on their research aims. However, in general, two studies stated significantly abnormal responses/worse scores in TBI, or concussion groups compared to control groups (62, 83) and increased sway, imbalance or abnormal results on one or more SOT scores were observed in two studies (49, 68). The other two studies reported no significant statistical differences within TBI groups (e.g., symptomatic versus asymptomatic TBI or those with normal versus saccular abnormalities) (51, 65). In one study, the dynamic posturography results were normal following TBI (43).

#### 3.3.10 Other tests

Hennebert’s sign, which is a pressure-induced nystagmus resulting from changes in pressure applied to the external auditory canal, can be a finding that indicates semicircular canal dehiscence, Meniere’s disease or vestibulofibrosis (94, 95). Fistula Test is used to assess the integrity of the bony labyrinth (95). Fistula test was negative in three studies (26, 29, 42). A positive Hennebert’s sign was observed in one study (39). In three of these studies, it was indicated through assessments that the patients had perilymphatic fistula (29, 39, 42).

#### 3.3.11 PROMs

Symptoms, quality-of-life, functional status, experiences or satisfaction can be evaluated via PROMs (96). PROMs related to vestibular disorders were conducted in 14 studies (46, 51, 52, 54, 61–65, 67, 71, 72, 74, 93), of which 4 studies reported using two PROMs (46, 51, 62, 63). The most commonly used PROM was Dizziness Handicap Inventory (DHI) (13/14) (46, 51, 52, 61–65, 67, 71, 72, 74, 93). Two studies used the Neurobehavioural Symptom Inventory (NSI) (51, 62), whilst others used the space and motion discomfort-II (SMD-II) (63), Post-concussion Symptom Scale (PCSS) (54), and Vertigo Symptom Scale Short Form (VSS-SF) (46).

Following TBI/concussion, 11 studies observed impairment based on DHI (46, 51, 52, 61–63, 67, 71, 72, 74, 93). In 2 studies, only reported the mean score of DHI and did not provide any interpretation of the score (64, 65). However, in 5 out of 13 studies, group comparisons (e.g., pre-post, control, with and without vestibular agnosia, positive and negative BPPV, normal and abnormal saccular function) indicated no significant difference in DHI scores (52, 64, 65, 67, 71). In studies using other PROMs in addition to the DHI, impairments were detected, as shown in Table 2 (46, 51, 62, 63).

### 3.4 Effect of severity of non-blast related TBI on vestibular outcomes

Twenty-three studies have not clearly stated the severity of TBI (23/50) (24–27, 29–33, 36, 38–42, 44, 45, 47–49, 59, 60, 66). Of the remaining 27 studies (7 of which were case studies/series), 9 studies included mild TBI (28, 43, 46, 50, 55, 62, 63, 65, 93), 12 studies reported concussions (i.e., mild TBI) (34, 37, 52–54, 64, 71, 72, 74, 75, 82, 83), one observed moderate/severe TBI (35), one study included severe TBI (58). Two studies included a range from mild to severe TBI (51, 67), whilst two studies involved participants with mild and moderate TBI (61, 68) (see Table 1 for more details on severity).

In four studies that included various TBI severity groups, different assessments were conducted (51, 61, 67, 68), although three studies did not perform any statistical assessment regarding severity (51, 67, 68). In one case comparison interventional study, BPPV was detected significantly more in individuals with moderate TBI (61) and in another study, most individuals with BPPV had moderate to severe TBI (67). In final study, the severity of TBI at which BPPV occurred was not specified (68).

Of the 9 studies in which TBI severity was classified as mild, four were case studies (28, 43, 46, 50). In 2 out of the nine studies, normal peripheral and central vestibular function was observed (43, 46). Although one case study reported a normal result, it was unclear whether normal vestibular function was fully present, as only a VNG test was conducted (28). BPPV was identified in 5 studies following mild TBI (46, 50, 62, 63, 65), whilst in 5 studies, one of the possible diagnoses observed included sensory integration dysfunction (46, 62, 93), persistent sensorimotor impairment (63), or peripheral, central vestibulopathy (55).

Abnormal findings were obtained in at least one test or subtest in 12 studies involving concussion (34, 37, 52–54, 64, 71, 72, 74, 75, 82, 83). Of these 12 studies, two were case studies (34, 37). The most commonly used assessment was VOMS (5/12) (34, 53, 54, 74, 75). Among studies using dynamic positional tests, one identified both posterior and horizontal SCC BPPV (64), whilst the other found no BPPV post-concussion (52). Two studies reported no significant differences in vestibular assessments between pre- and post-concussion or between those with and without a history of concussion (52, 75), whilst three studies observed a significant increase in VOMS scores post-concussion or in patients with a concussion history compared to those without (53, 54, 74). Additionally, throughout concussion-related studies, abnormalities in vestibular function (34, 37, 72), saccular or vestibulocollic function abnormalities (82), vestibulo-oculomotor dysfunction (54), vestibular-vision interaction deficits (71), persistent chronic vestibulo-oculomotor symptom provocation (74), and impairments in the central integration of vestibular function (83) were observed. In the study reporting severe TBI, posterior SCC and bilateral BPPV were observed (58). In moderate/severe TBI, bilateral normal nystagmus was observed on caloric (35).

PROMs were not used in any study in the severe TBI group. In the studies that were used, the results were reported differently from each other and the score severity for DHI, which was the most frequently used in the studies, was not stated consistently in each study. However, both mild TBI and concussion were reported impairments ranging from moderate to severe (46, 74). Furthermore, in studies with various TBI groups from mild to severe, one reported a mild impairment (51) while the other stated a moderate impairment in DHI (67).

In summary, the impact of TBI severity on vestibular outcomes varies across studies. Different outcomes can be observed for each TBI severity, from normal vestibular function to sensory integration dysfunction. In addition, due to differences in the reporting of DHI results and methodological approaches among the studies, a common conclusion could not be reached on the effect of TBI severity on DHI outcome.

### 3.5 Effect of aetiology of non-blast related TBI on vestibular outcomes

To investigate the impact of different aetiologies associated with TBI, they were categorised into five groups: falls, motor vehicle accidents (MVA), sports-related injuries, assaults, and others. Although penetrating TBI was not explicitly reported as an aetiology in the included studies, cases with potentially penetrating mechanisms may be present within the “other” category (e.g., industrial injuries involving metal impact (45)). The majority of studies (26/50) reported falls as the cause (24, 25, 30, 31, 38–40, 42, 44, 46, 48–51, 58–68, 93), followed by 21 studies reporting MVA (26–29, 32, 35, 36, 38, 43, 46, 49, 58, 59, 61–64, 66–68, 93). Across these aetiologies, a wide range of vestibular impairments were observed; however, BPPV was associated across all aetiologies except those including only sports-related TBI/concussion and was particularly common following falls.

In 15 studies reporting at least one TBI related to falls (26/50), at least one vestibular test showed abnormal results (24, 25, 30, 31, 38–40, 42, 44, 46, 48–50, 60, 65). Posterior, horizontal or anterior SCC BPPV due to fall was observed (25, 30, 40, 46, 50, 60, 65). In 4 studies, BPPV was observed only in the posterior SCC (30, 40, 50, 65); in 2 studies, it was found in both the posterior and horizontal SCCs (25, 46), and in one study, it was identified in both the anterior and posterior SCCs (60). There were 5 studies in which caloric testing showed absent bilateral or unilateral responses (24, 42, 44, 48, 49), while three studies showed normal caloric responses following a fall (31, 38, 44). However, in two studies where normal results were obtained, abnormal caloric results were detected in the follow-up assessment (31, 38).

Across studies reporting MVA (21/50), the most commonly used vestibular assessments were the caloric test (*n* = 6) (26, 27, 35, 36, 38, 49) or ENG/VNG (*n* = 5) (28, 29, 32, 38, 43). Similar to findings following falls, a range of results was observed in caloric testing following MVA in the first assessment, from normal (26, 35) responses to unilateral/bilateral abnormalities (27, 36, 49) or central tonus differences (38). Additionally, both normal (28, 43) and abnormal results were recorded in VNG/ENG (29, 32, 38). BPPV (38) or benign positional vertigo (32) was observed in two studies. In one study, it supported normal peripheral and central vestibular function in all tests (43).

In 11 studies reporting sports-related concussion or TBI, various test batteries were used. However, the commonly applied assessment was the VOMS (5/11) (53–55, 74, 75). In all of these studies, abnormal results were obtained in at least one VOMS subtest. Moreover, three studies identified significant differences between groups pre- and post-TBI (53, 54) or between those with and without a concussion history (74). No study reported BPPV in studies that only included sports-related TBI/concussion. However, positional tests were performed in only one study (52). Furthermore, various outcomes were observed across studies, ranging from vestibular dysfunction (72), abnormalities in saccular or vestibulocollic function (82), peripheral vestibular deficits (71), central or peripheral vestibulopathy (55) or impaired central integration of vestibular function despite a normal peripheral vestibular system (83).

Out of 4 studies reporting different types of assaults, normal SCC response (33), unilateral vestibular dysfunction (41, 42), and somatosensory integration dysfunction (46) were detected. Three studies were classified under “other” causes of injury, including striking the back of the head (42), being hit by a volleyball during practice (34), and an object falling from a bookcase (47). Of these three studies, one reported normal vestibular finding (47), whilst the other two reported abnormal vestibular findings (34, 42), including nystagmus on VOMS or spontaneous right-beating nystagmus.

There were 11 studies that included participants with a variety of aetiologies, from falls to assault (51, 58, 59, 61–64, 66–68, 93) (results reported together under all aetiologies). In 5 out of the eleven studies, results of each participant were reported separately, BPPV was observed following TBI due to both MVA and falls (59, 61, 64, 67, 68) (Table 2). Additionally, in one study, BPPV was observed following TBI due to MVA (58), whilst in another study, BPPV was reported but results were not categorised by different aetiologies (e.g., MVA, falls and blow to head) (66). In all studies reporting the affected SCC in cases of BPPV, the posterior SCC was the most impacted following both MVA and falls (59, 64). McCormick and Kolar (64) reported that falls were a statistically significant TBI aetiology for BPPV. In addition, another study reported that falls were a common aetiology among participants with vestibular hypofunction (68).

Although PROMs were utilised in studies examining various TBI aetiologies, the results were not separately reported by aetiology across those studies. Therefore, the effect of aetiology on PROMs could only be assessed in studies focusing on a single aetiology. No PROMs were used in the studies reporting MVA. In studies reporting falls, one study observed moderate and severe impairments (46), whilst studies associated with sports-related injuries identified mild (52) and severe impairments (74) based on DHI scores.

Summarily, while BPPV is commonly observed, particularly due to falls, common or different vestibular findings were detected across various aetiologies, including peripheral and central vestibular dysfunctions, abnormal ocular or postural responses, and impairments in sensory integration.

### 3.6 Effect of gender on vestibular outcomes following non-blast related TBI

Out of the 50 studies, 21 included both genders (38, 46, 49, 51, 53–55, 58, 59, 61–68, 72, 74, 83, 93), whilst the remaining studies either reported only male participants (n18) (24, 25, 27, 30, 31, 33, 36, 37, 39, 41–45, 48, 60, 71, 75), or only female participants (n10) (26, 28, 29, 32, 34, 35, 40, 47, 50, 82). One study did not specify gender (52).

In 7 out of the 21 studies that included both genders, multiple participants were included, but no results were reported by gender (54, 55, 62, 63, 65, 83, 93). Six out of these 7 studies had more males than females following TBI (54, 55, 63, 65, 83, 93). In the remaining 14 studies, although there were studies with multiple participants, either a statistical analysis was conducted between genders (51, 53, 68, 72, 74), results were presented based on gender in at least one assessment (58, 59, 61, 64, 66, 67), or in case studies, results were reported individually for each patient (38, 46, 49). A statistically significant difference was found for females in smooth pursuit, horizontal, and vertical saccades in the VOMS assessment (53), whilst Smulligan et al. (74) reported no statistically significant relationship between gender and VOMS performance. In two studies with similar vestibular assessments, there was no significant difference between vestibular function abnormalities and gender (68), nor was vestibular dysfunction correlated with gender (72). Moreover, there was no statistically significant relationship between vestibular score and gender in SOT (51). In studies where separate results were obtained, including case studies by gender, BPPV was observed in both males and females (38, 46, 49, 58, 59, 61, 64, 67). However, in 4 out of 8 studies, the number of males with BPPV was higher than the number of females (58, 59, 61, 67). Furthermore, in Ahn et al. (59) study, the number of males with posterior SCC BPPV was higher than females, while in another study, no males were observed with posterior SCC BPPV (64). However, Kim et al. (66) also reported that posterior SCC BPPV was more prevalent in males than females following TBI. Additionally, in all the studies included in this scoping review, the SCCs in which BPPV was observed were not consistently specified (Table 2).

In studies reporting male participants only, in 9 out of 18 studies, the caloric test revealed either a normal response (31, 44) or bilateral (24, 31, 42, 48) and unilateral abnormalities (27, 36, 41, 42, 44, 45). Furthermore, in three studies including only male participants where positional tests were applied, right posterior SCC BPPV was observed (25, 30, 60), while horizontal (25) or anterior SCCs (60) BPPV accompanied posterior SCC in two of these studies. Of the 10 studies including only female participants, normal horizontal SCC function via caloric test was identified in three studies (26, 35, 47). Similarly, in studies involving only females, bilateral or unilateral posterior SCC BPPV (40, 50) and benign positional vertigo (32) were reported. Moreover, following TBI, a range of vestibular outcomes were identified across genders. In males, findings ranged, from normal SCC function (e.g., normal HIT) (33) or overall peripheral and central vestibular functions (43) to perilymphatic fistula (39, 42) or vestibular-visual interaction deficits (37, 71, 75). In females, reported impairments included perilymphatic fistula (29), vestibulo-oculomotor dysfunction (34), and impairments in saccular or vestibulocollic function (e.g., abnormal c-VEMP responses) (82).

None of the studies that included only females used PROMs. In the study with only males, although the previous concussion group had a wider score range, there was no statistically significant difference between the comparison group (71). Among the 12 studies that included both genders, only 3 reported PROMs by gender (46, 72, 74). In two studies, there was no statistically significant association between gender and DHI (74) or that gender was not correlated with DHI score (72). In the case study, severe impairment was reported in male patients, while severe or moderate impairment was observed in females (46).

In summary, a variety of vestibular impairments, including BPPV, perilymphatic fistula, and vestibulo-oculomotor dysfunction, were observed in both male and female participants across different studies.

## 4 Discussion

This scoping review synthesised the common vestibular impairments associated with non-blast related TBI and investigated the influence of TBI severity, aetiology, and gender on vestibular outcomes. We found large inconsistencies in the reporting of vestibular tests, results and demographics across the studies, which not only highlight methodological and clinical standardisation deficiencies, but also complicate the understanding of the overall impact of TBI on the vestibular system.

In this review, the most commonly detected peripheral vestibular deficit following TBI was BPPV, most frequently involving the posterior SCC (15, 97, 98), which aligns with the anatomical predisposition for otoconia to accumulate in this canal due to gravity (99). In accordance with the existing literature, we also found that TBI can cause damage to other peripheral vestibular structures, including the labyrinth, vestibular nerve, and otolith organs (15, 100). In contrast to the findings reported here, Akin et al. (101) found in their review that otolith organs may be more damaged compared to SSCs following TBI. However, this discrepancy may be due to differences in the inclusion criteria of the reviews, for example, Akin et al. (101) included blast-related TBI. Therefore, a comprehensive assessment of all vestibular components in TBI patients is essential to draw a definitive conclusion regarding the relative susceptibility of otolith organs and SSCs to damage following TBI.

Some studies yielded important findings regarding the central contributions to the vestibular system. For example, Taylor et al. (68) reported that patients with abnormal oculomotor function following chronic TBI had significantly more difficulty in vestibular-dependent conditions on the SOT. Other research highlighted that, despite normal oculomotor and peripheral vestibular function following chronic TBI, a high proportion of abnormal SOT performance was still observed (62). This review also identified that patients with normal peripheral vestibular function who exhibited vestibular agnosia (impaired self-motion perception), and along with acute TBI patients, showed greater posturography instability than those without vestibular agnosia (67). All these findings support the view that balance relies not only on the inner ear and brainstem pathways but also on cortical processing and integration of vestibular signals (102). Moreover, self-motion perception is considered to arise from the integration of multiple brain regions, rather than being localised to a single region (67).

Similar to vestibular tests, the lack of standardisation in the application of PROMs and the reporting of results was observed throughout the records. Although the DHI, which was more commonly used than other PROMs, identified impairments, findings from some studies indicate an inability to differentiate between different groups. This may be an indication that the DHI may not be an appropriate measure of vestibular symptoms and quality-of-life for this specific patient population. To our knowledge, there is no specific study on the validity and reliability of using the DHI for adults with TBI. However, a study investigating the clinical utility of the DHI for Children and Adolescents (DHI-CA) (103) post-concussion reported that its clinical utility was questionable (104). In this context, developing specific PROMs especially for vestibular impairments in adults with TBI or determining which of the existing PROMs is more suitable in this population can be important for evaluating the quality-of-life and monitoring rehabilitation processes.

The observation of both peripheral vestibular impairments and central impairments across different TBI severities, including mild TBI, may suggest that TBI severity does not necessarily influence vestibular outcomes. Similarly, BPPV was reported across all severities, although some studies found BPPV more frequently in moderate (105) or severe TBI (58) than in mild TBI. Remarkably, the finding that even after concussion, central processing of vestibular and visual information may be altered without affecting the peripheral vestibular organs or associated brainstem and cerebellar processes (83) underscores the importance of a comprehensive evaluation in all TBI severities.

The findings suggest that the aetiology of TBI may be associated with the presence of BPPV, influence of aetiology does not follow a clear pattern on other peripheral and central vestibular impairments. Although BPPV was commonly reported following fall in our review, in contrast, MVA was also reported to be one of the most frequent causes of BPPV in the literature (98, 106). Therefore, further studies specifically designed to understand the impact of TBI aetiology on vestibular findings are needed.

The observation of similar peripheral or central vestibular outcomes in both males and females, along with differing statistical results from included studies, made it difficult to draw conclusions about the gender differences. Regarding BPPV, the findings suggest that BPPV may be more common in males, whilst there are studies in the literature that report no gender difference in BPPV following TBI (106, 107). Additionally, in line with the results of Teramoto et al. (53), a few studies were found statistically significant higher impairment in females compared to males in some oculomotor and vestibular assessments (108, 109). However, since these studies were mainly conducted with adolescents and those experiencing sport-related concussions, the generalisability of these results to the entire adult TBI population is limited.

The timing of assessment can have a significant impact on the interpretation of vestibular outcomes following TBI. Although the post-injury assessment times were extracted in the included studies, the results were not analysed according to assessment time. The terms “acute” and “chronic” TBI are commonly used in the literature, whilst there is no consistent agreement regarding the exact time frames for these periods. For example, some of the included studies accepted the acute period to last up to approximately 3 months post-injury (67, 68), whereas other extended this period to 6 months (74). Future research should clearly define different TBI phases such as acute, subacute, chronic, or post-chronic, and stratify vestibular outcomes accordingly. This would allow investigation of whether the effects of certain variables differ over time and contribute to the development of more effective assessment and management strategies. Moreover, future studies should also investigate which vestibular tests and assessment protocols are most appropriate at different post-injury time points to provide guidance for clinical practice.

Our findings provide insights into the common peripheral and central vestibular impairments following non-blast related TBI, while highlighting the complexity of understanding the effects of factors such as severity, aetiology, and gender due to the multifaceted nature of vestibular deficits combined with the intricate nature of TBI. Future research should develop comprehensive vestibular assessment protocols for individuals with TBI and focus on consistent methodology and standardised reporting of results to better understand the effects of variables on vestibular findings.

### 4.1 Strengths and limitations

This scoping review study provided a comprehensive review of the literature related to the research questions. Through the established inclusion criteria, studies that precisely matched the definition of TBI were carefully selected to ensure the examination of TBI-specific findings. However, the differences across research questions and the heterogeneity in the study designs of the included studies (for example, high proportion of case studies/series) have complicated the synthesis and comparison of the findings. Furthermore, the assessment times reported following TBI (e.g., acute and chronic TBI) were widely variable between records, with some not reporting the assessment time clearly. Moreover, the assessment time for acute TBI is debated in the literature and therefore, it was not possible to group the data into acute and chronic TBI and as such we were unable to investigate the effect of assessment time. Additionally, due to the broad scope of the study, some vestibular findings accompanied by auditory findings in certain studies were addressed in another scoping review. Although including only English-language studies may have limited the results, the inclusion of studies from non-English-speaking countries allowed for the presentation of results from a broader perspective.

## 5 Conclusion

Our review has demonstrated the diversity of vestibular findings following non-blast related TBI. However, the complexities of the vestibular system and TBI, as well as inconsistencies in vestibular assessment methods and reporting approaches (e.g., lack of clear specification of oculomotor assessment method) and lack of consistent use of PROMs, limit a comprehensive understanding of vestibular findings in individuals with TBI. These limitations hinder the ability to identify which methods should be prioritised for vestibular assessment in individuals with TBI and, consequently, obstruct the development of diagnostic and therapeutic processes. In this context, future research should focus on adopting more consistent methodologies and standardised reporting practices to enhance vestibular assessment and management approaches for individuals with TBI. To achieve this, establishing some level of agreement or consensus on testing protocols can be beneficial. Additionally, investigating the effects of variables such as severity, aetiology, and gender on vestibular findings through large-scale studies is important for developing more effective interventions for this patient group.

## In memoriam

We would like to express our gratitude to Professor David Baguley, who sadly passed away during this study. His knowledge and expertise helped develop the fundamental ideas for this research, for which we are very thankful.

## Glossary

BPPV: Benign Paroxysmal Positional Vertigo
c-VEMP: cervical VEMP
DHI: Dizziness Handicap Inventory
DVA: Dynamic Visual Acuity
ENG: Electronystagmography
GCS: Glasgow Coma Scale
GST: Gaze Stabilization Test
mCTSIB: modified Clinical Test of Sensory Integration and Balance
MVA: Motor Vehicle Accidents
NSI: Neurobehavioural Symptom Inventory
o-VEMP: ocular VEMP
PCSS: Post-Concussion Symptom Scale
PROMs: Patient-Reported Outcome Measurements
SCCs: Semicircular Canals
SMD-II: Space and Motion Discomfort-II
SOT: Sensory Organization Test
TBI: Traumatic Brain Injury
UK: United Kingdom
VEMP: Vestibular Evoked Myogenic Potential
vHIT: video Head Impulse Test
VNG: Videonystagmography
VOMS: Vestibular-Oculomotor Screening
VSS-SF: Vertigo Symptom Scale Short Form
VVOR: Visual Vestibulo-Ocular Reflex
VORS: Vestibulo-Ocular Reflex Suppression

## References

[1] Dewan MC Rattani A Gupta S Baticulon RE Hung YC Punchak M . Estimating the global incidence of traumatic brain injury. J Neurosurg. (2019) 130:1080–97. doi: 10.3171/2017.10.JNS17352, 29701556

[2] Capizzi A Woo J Verduzco-Gutierrez M. Traumatic brain injury: an overview of epidemiology, pathophysiology, and medical management. Medical Clinics. (2020) 104:213–38. doi: 10.1016/j.mcna.2019.11.001, 32035565

[3] Benedictus MR Spikman JM Van Der Naalt J. Cognitive and behavioral impairment in traumatic brain injury related to outcome and return to work. Arch Phys Med Rehabil. (2010) 91:1436–41. doi: 10.1016/J.APMR.2010.06.019, 20801264

[4] Khoury S Benavides R. Pain with traumatic brain injury and psychological disorders. Prog Neuro-Psychopharmacol Biol Psychiatry. (2018) 87:224–33. doi: 10.1016/J.PNPBP.2017.06.007, 28627447

[5] Kornblith ES Langa KM Yaffe K Gardner RC. Physical and functional impairment among older adults with a history of traumatic brain injury. J. Head Trauma Rehabil. (2020) 35:E320–9. doi: 10.1097/HTR.0000000000000552, 31996604 PMC7335322

[6] Humphreys I Wood RL Phillips CJ Macey S. The costs of traumatic brain injury: a literature review. ClinicoEcon Outcomes Res. (2013) 5:281–7. doi: 10.2147/CEOR.S44625, 23836998 PMC3699059

[7] Ahmed Z. Current clinical trials in traumatic brain injury. Brain Sci. (2022) 12. doi: 10.3390/brainsci12050527, 35624914 PMC9138587

[8] Teasdale G Jennett B. Assessment of coma and impaired consciousness: a practical scale. Lancet. (1974) 304:81–4. doi: 10.1016/S0140-6736(74)91639-0 4136544

[9] Maas AIR Menon DK Manley GT Abrams M Åkerlund C Andelic N . Traumatic brain injury: progress and challenges in prevention, clinical care, and research. Lancet Neurol. (2022) 21:1004–60. doi: 10.1016/S1474-4422(22)00309-X, 36183712 PMC10427240

[10] de Lanerolle Jung H Bandak Faris A. Neuropathology of traumatic brain injury: comparison of penetrating, nonpenetrating direct impact and explosive blast etiologies. Semin Neurol. (2015) 35:12–9. doi: 10.1055/s-0035-1544240, 25714863

[11] Maskell F Chiarelli P Isles R. Dizziness after traumatic brain injury: overview and measurement in the clinical setting. Brain Inj. (2006) 20:293–305. doi: 10.1080/02699050500488041, 16537271

[12] Alsalaheen BA Whitney SL Mucha A Morris LO Furman JM Sparto PJ. Exercise prescription patterns in patients treated with vestibular rehabilitation after concussion. Physiother Res Int. (2013) 18:100–8. doi: 10.1002/pri.1532, 22786783 PMC4894842

[13] Wallace B Lifshitz J. Traumatic brain injury and vestibulo-ocular function: current challenges and future prospects. Eye Brain. (2016) 8:153–64. doi: 10.2147/EB.S82670, 28539811 PMC5398755

[14] Kolev O Sergeeva M. Vestibular disorders following different types of head and neck trauma. Funct Neurol. (2015) 31:1–6. doi: 10.11138/FNeur/2016.31.2.075, 27358219 PMC4936800

[15] Šarkić B Douglas JM Simpson A Vasconcelos A Scott BR Melitsis LM . Frequency of peripheral vestibular pathology following traumatic brain injury: a systematic review of literature. Int J Audiol. (2021) 60:479–94. doi: 10.1080/14992027.2020.1811905, 32907431

[16] Smith RM Marroney N Beattie J Newdick A Tahtis V Burgess C . A mixed methods randomised feasibility trial investigating the management of benign paroxysmal positional vertigo in acute traumatic brain injury. Pilot Feasibility Stud. (2020) 6:130. doi: 10.1186/s40814-020-00669-z, 32944278 PMC7493395

[17] Peters MDJ Godfrey CM Khalil H McInerney P Parker D Soares CB. Guidance for conducting systematic scoping reviews. Int J Evid Based Healthc. (2015) 13:141–6. doi: 10.1097/XEB.0000000000000050, 26134548

[18] Arksey H O’Malley L. Scoping studies: towards a methodological framework. Int J Soc Res Methodol Theory Practi. (2005) 8:19–32. doi: 10.1080/1364557032000119616

[19] Rethlefsen ML Kirtley S Waffenschmidt S Ayala AP Moher D Page MJ . Prisma-s: an extension to the PRISMA statement for reporting literature searches in systematic reviews. Syst Rev. (2021) 10. doi: 10.1186/s13643-020-01542-z, 34285662 PMC8270366

[20] Lefebvre C Glanville J Briscoe S Littlewood A Marshall C Metzendorf M-I . Searching for and selecting studies In: Higgins JPT Green S, editors. Cochrane handbook for systematic reviews of interventions. Version: The Cochrane Collaboration (2025). 6:5, Available online at: cochrane.org/handbook

[21] Higgins JP Lasserson T Chandler J Tovey D Churchill R. Methodological expectations of Cochrane intervention reviews. Cochrane: London. 2019.

[22] McGowan J Sampson M Salzwedel DM Cogo E Foerster V Lefebvre C. PRESS peer review of electronic search strategies: 2015 guideline; statement. J Clin Epidemiol. (2016) 75:40–6. doi: 10.1016/j.jclinepi.2016.01.021, 27005575

[23] Ouzzani M Hammady H Fedorowicz Z Elmagarmid A. Rayyan—a web and mobile app for systematic reviews. Syst Rev. (2016) 5:210. doi: 10.1186/s13643-016-0384-4, 27919275 PMC5139140

[24] Feneley MR Murthy P. Acute bilateral vestibulo-cochlear dysfunction following occipital fracture. J Laryngol Otol. (1994) 108:54–6. doi: 10.1017/S0022215100125836, 8133170

[25] Bertholon P Chelikh L Timoshenko AP Tringali S Martin C. Combined horizontal and Posterior Canal benign paroxysmal positional Vertigo in three patients with head trauma. Ann Otol Rhinol Laryngol. (2005) 114:105–10. doi: 10.1177/000348940511400204, 15757188

[26] Kagoya R Ito K Kashio A Karino S Yamasoba T. Dislocation of stapes with footplate fracture caused by indirect trauma. Ann Otol Rhinol Laryngol. (2010) 119:628–30. doi: 10.1177/000348941011900910, 21033031

[27] Ylikoski J. Palva T. Sanna M. Dizziness after head trauma: clinical and morphologic findings Am J Otol (1982) 3:343–352. 7081411

[28] Roup CM Ross C Whitelaw G. Hearing difficulties as a result of traumatic brain injury. J Am Acad Audiol. (2020) 31:137–46. doi: 10.3766/jaaa.18084, 31287053

[29] Fitzgerald DC. Persistent dizziness following head trauma and perilymphatic fistula. Arch Phys Med Rehabil. (1995) 76:1017–20. doi: 10.1016/S0003-9993(95)81041-2 7487449

[30] Ralli G Francesca A Antonio L Giuseppe N. Post-traumatic camel-related benign paroxysmal positional vertigo. Travel Med Infect Dis. (2010) 8:207–9. doi: 10.1016/j.tmaid.2010.07.002, 20970722

[31] Fujimoto C Ito K Takano S Karino S Iwasaki S. Successful cochlear implantation in a patient with bilateral progressive sensorineural hearing loss after traumatic subarachnoid hemorrhage and brain contusion. Ann Otol Rhinol Laryngol. (2007) 116:897–901. doi: 10.1177/000348940711601205, 18217508

[32] Ottaviano G Marioni G Marchese-Ragona R Trevisan CP De Filippis C Staffieri A. Anosmia associated with hearing loss and benign positional vertigo after head trauma. Acta Otorhinolaryngol Ital. (2009) 29:270–3. 20162029 PMC2821127

[33] Kanavati O Salamat AA Tan TY Hellier W. Bilateral temporal bone fractures associated with bilateral profound sensorineural hearing loss. Postgrad Med J. (2016) 92:302–3. doi: 10.1136/postgradmedj-2015-133862, 26719451

[34] Blackard MF Sawhney V Castillo M Gupta M Pandya AS Patel R. A case report of concussion in female collegiate volleyball player. Sports Orthop Traumatol. (2020) 36:377–83. doi: 10.1016/j.orthtr.2020.08.001

[35] Jani NN Laureno R Mark AS Brewer CC. Deafness after bilateral midbrain contusion: a correlation of magnetic resonance imaging with auditory brain stem evoked responses. Neurosurgery (1991) 29:106–9. 1870669

[36] Schuknecht HF Davison RC. Deafness and Vertigo from head injury. AMA Arch Otolaryngol. (1956) 63:513–28. doi: 10.1001/archotol.1956.03830110055006, 13312806

[37] Waninger KN Gloyeske BM Hauth JM Vanic KA Yen DM. Intratympanic hemorrhage and concussion in a football offensive lineman. J Emerg Med. (2014) 46:371–2. doi: 10.1016/j.jemermed.2013.08.043, 24161227

[38] Preber L Silversklöld BP. Paroxysmal positional Vertigo following head injury: studied by electronystagmography and skin resistance measurements. Acta Otolaryngol. (1957) 48:255–65. doi: 10.3109/00016485709124379, 13469324

[39] Sousa Menezes A Ribeiro D Miranda DA Martins Pereira S. Perilymphatic fistula and pneumolabyrinth without temporal bone fracture: a rare entity. BMJ Case Rep. (2019) 12:e228457. doi: 10.1136/bcr-2018-228457, 30826783 PMC6398632

[40] Lerut B De Vuyst C Ghekiere J Vanopdenbosch L Kuhweide R. Post-traumatic pulsatile tinnitus: the hallmark of a direct carotico-cavernous fistula. J Laryngol Otol. (2007) 121:1103–7. doi: 10.1017/S0022215107005890, 17295936

[41] Durbec M Vigier S Brosset R Mottier C Dubreuil C Tringali S. Post-traumatic total deafness with normal CT scan. Eur Ann Otorhinolaryngol Head Neck Dis. (2012) 129:281–3. doi: 10.1016/j.anorl.2011.12.004, 23073497

[42] Lyos AT Marsh MA Jenkins HA Coker NJ. Progressive hearing loss after transverse temporal bone fracture. Arch Otolaryngol Head Neck Surg. (1995) 121:795–9. doi: 10.1001/archotol.1995.01890070081017, 7598860

[43] Paxman E Stilling J Mercier L Debert CT. Repetitive transcranial magnetic stimulation (rTMS) as a treatment for chronic dizziness following mild traumatic brain injury. BMJ Case Rep. (2018) 2018:bcr-2018-226698. doi: 10.1136/bcr-2018-226698, 30396889 PMC6229180

[44] Tonkin JP Fagan P. Rupture of the round window membrane. J Laryngol Otol. (1975) 89:733–56. doi: 10.1017/S0022215100080944, 1176821

[45] Mohd Khairi MD Irfan M Rosdan S. Traumatic head injury with contralateral sensorineural hearing loss. Ann Acad Med Singap. (2009) 38:1017–8. doi: 10.47102/annals-acadmedsg.V38N11p1017, 19956827

[46] Kleffelgaard I Soberg HL Bruusgaard KA Tamber AL Langhammer B. Vestibular rehabilitation after traumatic brain injury: case series. Phys Ther. (2016) 96:839–49. doi: 10.2522/ptj.20150095, 26586860

[47] Jacobs GB Lehrer JF Rubin RC Hubbard JH Nalebuff DJ Wille RL. Posttraumatic vertigo: report of three cases. J Neurosurg. (1979) 51:860–1. doi: 10.3171/jns.1979.51.6.0860, 501429

[48] Chung JH Shin MC Min HJ Park CW Lee SH. Bilateral cochlear implantation in a patient with bilateral temporal bone fractures. Am J Otolaryngol. (2011) 32:256–8. doi: 10.1016/j.amjoto.2010.03.002, 20444523

[49] Herdman S. J. Treatment of vestibular disorders in traumatically brain-injured patients. J Head Trauma Rehabil (1990) 5:63–76. doi: 10.1097/00001199-199012000-00008

[50] Johnson EG. Clinical Management of a Patient with chronic recurrent Vertigo following a mild traumatic brain injury. Case Rep Med. (2009) 2009:910596. doi: 10.1155/2009/910596, 19826635 PMC2760235

[51] Joseph A-LC Lippa SM Moore B Bagri M Row J Chan L . Relating self-reported balance problems to sensory organization and dual-tasking in chronic traumatic brain injury. PM&R. (2021) 13:870–9. doi: 10.1002/pmrj.12478, 32844594 PMC10440855

[52] Hides JA Franettovich Smith MM Mendis MD Smith NA Cooper AJ Treleaven J . A prospective investigation of changes in the sensorimotor system following sports concussion. An exploratory study. Musculoskelet Sci Pract. (2017) 29:7–19. doi: 10.1016/j.msksp.2017.02.003, 28259770

[53] Teramoto M Grover EB Cornwell J Zhang R Boo M Ghajar J . Sex differences in common measures of concussion in college athletes. J Head Trauma Rehabil. (2022) 37:E299–309. doi: 10.1097/HTR.0000000000000732, 34698682

[54] Glendon K Blenkinsop G Belli A Pain M. Does vestibular-ocular-motor (VOM) impairment affect time to return to play, symptom severity, Neurocognition and academic ability in student-athletes following acute concussion? Brain Inj. (2021) 35:788–97. doi: 10.1080/02699052.2021.1911001, 33896286

[55] Uyeno C Zhang R Cornwell J Teramoto M Boo M Lumba-Brown A. Acute eye-tracking changes correlated with vestibular symptom provocation following mild traumatic brain injury. Clin J Sport Med. (2024) 34:411–6. doi: 10.1097/JSM.0000000000001223, 38702871

[56] Dix MR Hallpike CS. The pathology, symptomatology and diagnosis of certain common disorders of the vestibular system. Proc R Soc Med. (1952) 45:341–54. doi: 10.1177/003591575204500604, 14941845 PMC1987487

[57] BSA. Recommended Procedure Positioning Tests. (2016). 9–17. Available online at: www.thebsa.org (Accessed October 15, 2024).

[58] Motin M Keren O Groswasser Z Gordon CR. Benign paroxysmal positional vertigo as the cause of dizziness in patients after severe traumatic brain injury: diagnosis and treatment. Brain Inj. (2005) 19:693–7. doi: 10.1080/02699050400013600, 16195183

[59] Ahn S-K Jeon S-Y Kim J-P Park JJ Hur DG Kim D-W . Clinical characteristics and treatment of benign paroxysmal positional vertigo after traumatic brain injury. J Trauma Acute Care Surg. (2011) 70:442–6. doi: 10.1097/TA.0b013e3181d0c3d9, 20489667

[60] Dlugaiczyk J Siebert S Hecker DJ Brase C Schick B. Involvement of the anterior semicircular canal in posttraumatic benign paroxysmal positioning vertigo. Otol Neurotol. (2011) 32:1285–90. doi: 10.1097/MAO.0b013e31822e94d9, 21892120

[61] Ouchterlony D Masanic C Michalak A Topolovec-Vranic J Rutka JA. Treating benign paroxysmal positional vertigo in the patient with traumatic brain injury: effectiveness of the canalith repositioning procedure. J Neurosci Nurs. (2016) 48:90–9. doi: 10.1097/JNN.0000000000000186, 26895567

[62] Campbell KR Parrington L Peterka RJ Martini DN Hullar TE Horak FB . Exploring persistent complaints of imbalance after mTBI: oculomotor, peripheral vestibular and central sensory integration function. J Vestib Res. (2021) 31:519–30. doi: 10.3233/VES-201590, 34024798 PMC12579460

[63] Galea O O’Leary S Williams K Treleaven J. Investigation of sensorimotor impairments in individuals 4 weeks to 6 months after mild traumatic brain injury. Arch Phys Med Rehabil. (2022) 103:921–8. doi: 10.1016/j.apmr.2021.10.029, 34861233

[64] McCormick K Kolar B. Research letter: rate of BPPV in patients diagnosed with concussion. J Head Trauma Rehabil. (2023) 38:434–8. doi: 10.1097/HTR.0000000000000867, 36854138

[65] Jafarzadeh S Pourbakht A Bahrami E. Vestibular assessment in patients with persistent symptoms of mild traumatic brain injury. Indian J Otolaryngol Head Neck Surg. (2022) 74:272–80. doi: 10.1007/s12070-020-02043-0, 36032895 PMC9411379

[66] Kim C-H Kim H Jung T Lee D-H Shin JE. Clinical characteristics of benign paroxysmal positional vertigo after traumatic brain injury. Brain Inj. (2024) 38:341–6. doi: 10.1080/02699052.2024.2310790, 38297437

[67] Calzolari E Chepisheva M Smith RM Mahmud M Hellyer PJ Tahtis V . Vestibular agnosia in traumatic brain injury and its link to imbalance. Brain. (2021) 144:128–43. doi: 10.1093/brain/awaa386, 33367536 PMC7880674

[68] Taylor RL Wise KJ Taylor D Chaudhary S Thorne PR. Patterns of vestibular dysfunction in chronic traumatic brain injury. Front Neurol. (2022) 13. doi: 10.3389/fneur.2022.942349, 36530624 PMC9751886

[69] Doettl SM McCaslin DL. Oculomotor assessment in children. Semin Hear. (2018) 39:275–87. doi: 10.1055/s-0038-1666818, 30038455 PMC6054585

[70] González JE Kiderman A. ENG/VNG In: Kountakis SE, editor. Encyclopedia of otolaryngology, head and neck surgery. Berlin, Heidelberg: Springer Berlin Heidelberg (2013). 785–92.

[71] Honaker JA Criter RE Patterson JN Jones SM. Gaze stabilization test asymmetry score as an indicator of previous concussion in a cohort of collegiate football players. Clin J Sport Med. (2015) 25:361–366. doi: 10.1097/JSM.0000000000000138, 25061806

[72] Gard A Al-Husseini A Kornaropoulos EN De Maio A Tegner Y Björkman-Burtscher I . Post-concussive vestibular dysfunction is related to injury to the inferior vestibular nerve. J Neurotrauma. (2022) 39:829–40. doi: 10.1089/neu.2021.0447, 35171721 PMC9225415

[73] Mucha A Collins MW Elbin RJ Furman JM Troutman-Enseki C DeWolf RM . A brief vestibular/ocular motor screening (VOMS) assessment to evaluate concussions: preliminary findings. Am J Sports Med. (2014) 42:2479–86. doi: 10.1177/0363546514543775, 25106780 PMC4209316

[74] Smulligan KL Carry P Smith AC Esopenko C Baugh CM Wilson JC . Cervical spine proprioception and vestibular/oculomotor function: an observational study comparing young adults with and without a concussion history. Phys Ther Sport. (2024) 69:33–9. doi: 10.1016/j.ptsp.2024.07.002, 39013262 PMC11343652

[75] Brown DA Grant G Evans K Leung FT Hides JA. Evaluation of the vestibular/ocular motor screening assessment in active combat sport athletes: an exploratory study. Brain Inj. (2022) 36:961–7. doi: 10.1080/02699052.2022.2109741, 35943357

[76] Hallpike CS. The caloric tests. J Laryngol Otol. (1956) 70:15–28. doi: 10.1017/S0022215100052610, 13278645

[77] Halmagyi GM Chen L MacDougall HG Weber KP McGarvie LA Curthoys IS. The video head impulse test. Front Neurol. (2017) 8. doi: 10.3389/fneur.2017.00258, 28649224 PMC5465266

[78] Halmagyi GM Curthoys IS. A clinical sign of canal paresis. Arch Neurol. (1988) 45:737–9. doi: 10.1001/archneur.1988.00520310043015, 3390028

[79] Hall S. F. Laird M. E. Is head-shaking nystagmus a sign of vestibular dysfunction? J Otolaryngol (1992) 21:209–212. 1404573

[80] Welgampola MS Colebatch JG. Characteristics and clinical applications of vestibular-evoked myogenic potentials. Neurology. (2005) 64:1682–8. doi: 10.1212/01.WNL.0000161876.20552.AA, 15911791

[81] Colebatch JG Rosengren SM Welgampola MS. Chapter 10 – Vestibular-evoked myogenic potentials In: Furman JM Lempert T, editors. Handbook of clinical neurology: Elsevier (2016). 133–55. 10.1016/B978-0-444-63437-5.00010-8 27638068

[82] Felipe L Shelton JA. The clinical utility of the cervical vestibular-evoked myogenic potential (cVEMP) in university-level athletes with concussion. Neurol Sci. (2021) 42:2803–9. doi: 10.1007/s10072-020-04849-w, 33161456

[83] Christy JB Cochrane GD Almutairi A Busettini C Swanson MW Weise KK. Peripheral vestibular and balance function in athletes with and without concussion. J Neurol Phys Ther. (2019) 43:153–9. doi: 10.1097/NPT.0000000000000280, 31205229 PMC6590702

[84] Zalewski CK McCaslin DL Carlson ML. Rotary chair testing In: Babu S Schutt CA Bojrab DI, editors. Diagnosis and treatment of vestibular disorders. Cham: Springer International Publishing (2019). 75–98.

[85] Van EAAJ Groen JJ Jongkees LBW. The turning test with small Regulable stimuli. J Laryngol Otol. (1948) 62:63–9. doi: 10.1017/S0022215100008690, 18900876

[86] Kobel MJ Wagner AR Merfeld DM Mattingly JK. Vestibular thresholds: a review of advances and challenges in clinical applications. Front Neurol. (2021) 12:643634. doi: 10.3389/fneur.2021.643634, 33679594 PMC7933227

[87] Seemungal B Gunaratne IA Fleming I Gresty MA Bronstein A. Perceptual and nystagmic thresholds of vestibular function in yaw. J Vestib Res. (2004) 14:461–6. doi: 10.3233/VES-2004-14604, 15735328

[88] Herdman SJ Tusa RJ Blatt P Suzuki A Venuto PJ Roberts D. Computerized dynamic visual acuity test in the assessment of vestibular deficits. Am J Otol (1998) 19:790–96. doi: 10.1016/S1567-4231(10)09014-3 9831156

[89] Goebel JA Tungsiripat N Sinks B Carmody J. Gaze stabilization test: a new clinical test of unilateral vestibular dysfunction. Otol Neurotol. (2007) 28:68–73. doi: 10.1097/01.mao.0000244351.42201.a7, 17106431

[90] Baloh RW Jacobson KM Beykirch K Honrubia V. Static and dynamic posturography in patients with vestibular and cerebellar lesions. Arch Neurol. (1998) 55:649–54. doi: 10.1001/archneur.55.5.649, 9605721

[91] Vališ M Dršata J Kalfeřt D Semerák P Kremláček J. Computerised static posturography in neurology. Open Med. (2012) 7:317–22. doi: 10.2478/s11536-011-0152-8

[92] Bronstein AM Pavlou M. Chapter 16 – Balance In: Barnes MP Good DC, editors. Handbook of clinical neurology: Amsterdam Elsevier (2013). 189–208. 10.1016/B978-0-444-52901-5.00016-2 23312641

[93] Lin L-F Liou T-H Hu C-J Ma H-P Ou J-C Chiang Y-H . Balance function and sensory integration after mild traumatic brain injury. Brain Inj. (2015) 29:41–6. doi: 10.3109/02699052.2014.955881, 25265292

[94] Hennebert C. A new syndrome in her editary syphilis of the labyrinth. Presse Med Belg Brux (1911) 63:467–470. Available online at: https://cir.nii.ac.jp/crid/1573387449164406400.bib?lang=ja (Accessed October 29, 2024)

[95] Pearlman RC. The fistula and Hennebert tests. J Am Audiol Soc. (1976) 2:1–2. 965273

[96] Devlin JN Appleby J. Getting the most out of PROMs: Putting health outcomes at the heart of NHS decision-making. London: King’s Fund (2010). 83 p.

[97] Carr S Rutka J. Post-traumatic dizziness. Curr Otorhinolaryngol Rep. (2017) 5:142–51. doi: 10.1007/s40136-017-0154-4

[98] Haripriya GR Mary P Dominic M Goyal R Sahadevan A. Incidence and treatment outcomes of post traumatic BPPV in traumatic brain injury patients. Indian J Otolaryngol Head Neck Surg. (2018) 70:337–41. doi: 10.1007/s12070-018-1329-0, 30211085 PMC6127059

[99] Parnes LS Agrawal SK Atlas J Diagnosis and management of benign paroxysmal positional vertigo (BPPV) Can Med Assoc J (2003) 169:681–93. 14517129 PMC202288

[100] Knoll RM Ishai R Trakimas DR Chen JX Nadol J Jb . Peripheral vestibular system histopathologic changes following head injury without temporal bone fracture. Otolaryngol Head Neck Surg. (2019) 160:122–30. doi: 10.1177/0194599818795695 30274548

[101] Akin FW Murnane OD Hall CD Riska KM. Vestibular consequences of mild traumatic brain injury and blast exposure: a review. Brain Inj. (2017) 31:1188–94. doi: 10.1080/02699052.2017.1288928, 28981340

[102] Hadi Z Mahmud M Seemungal BM. Brain mechanisms explaining postural imbalance in traumatic brain injury: a systematic review. Brain Connect. (2024) 14:144–77. doi: 10.1089/brain.2023.0064, 38343363

[103] Sousa M da GC de Cruz O Santos AN Ganança C Almeida L Sena EP de Brazilian adaptation of the dizziness handicap inventory for the pediatric population: reliability of the results Audiol. Commun. Res. 2015 20 327–335 doi: 10.1590/2317-6431-2015-1595

[104] Tiwari D Gochyyev P. Does the dizziness handicap inventory—children and adolescents (DHI-CA) demonstrate properties to support clinical application in the post-concussion population: a Rasch analysis. Children. (2023) 10:14. doi: 10.3390/children10091428, 37761389 PMC10528530

[105] Andersson H Jablonski GE Nordahl SHG Nordfalk K Helseth E Martens C . The risk of benign paroxysmal positional Vertigo after head trauma. Laryngoscope. (2022) 132:443–8. doi: 10.1002/lary.29851, 34487348

[106] Gordon CR Levite R Joffe V Gadoth N. Is posttraumatic benign paroxysmal positional Vertigo different from the idiopathic form? Arch Neurol. (2004) 61:1590–3. doi: 10.1001/archneur.61.10.1590, 15477514

[107] Di Cesare T Tricarico L Passali GC Sergi B Paludetti G Galli J . Traumatic benign paroxysmal positional vertigo: personal experience and comparison with idiopathic BPPV. Int J Audiol. (2021) 60:393–7. doi: 10.1080/14992027.2020.1821253, 32959692

[108] Sufrinko AM Mucha A Covassin T Marchetti G Elbin RJ Collins MW . Sex differences in vestibular/ocular and neurocognitive outcomes after sport-related concussion. Clin J Sport Med. (2017) 27:133–8. doi: 10.1097/JSM.0000000000000324, 27379660 PMC5203982

[109] Gray M Wilson JC Potter M Provance AJ Howell DR. Female adolescents demonstrate greater oculomotor and vestibular dysfunction than male adolescents following concussion. Phys Ther Sport. (2020) 42:68–74. doi: 10.1016/j.ptsp.2020.01.001, 31935640 PMC9258790

[110] The Management and Rehabilitation of Post-Acute mTBI Work Group. VA/DoD clinical practice guideline for the management and rehabilitation of post-acute mild traumatic brain injury. (2021). Available online at: www.tricare.mil (Accessed October 25, 2024).

[111] McCrory P Meeuwisse W Dvorak J Aubry M Bailes J Broglio S . Consensus statement on concussion in sport-the 5th international conference on concussion in sport held in Berlin, October 2016. Br J Sports Med. (2017) 51:838. doi: 10.1136/bjsports-2017-097699 28446457

[112] Malec JF Brown AW Leibson CL Flaada JT Mandrekar JN Diehl NN . The Mayo classification system for traumatic brain injury severity. J Neurotrauma. (2007) 24:1417–24. doi: 10.1089/neu.2006.0245, 17892404

